# Genetic/epigenetic RNA dysregulation in type 2 *diabetes mellitus* complicated with ischemic heart disease

**DOI:** 10.3389/fendo.2025.1687145

**Published:** 2025-10-31

**Authors:** Eslam S. Sedkey, Marwa Matboli, Mohamed G. Seadawy, Marwa G.A. Hegazy

**Affiliations:** ^1^ Chemical Warfare Department, Ministry of Defense, Cairo, Egypt; ^2^ Biochemistry Department, Faculty of Science, Ain Shams University, Cairo, Egypt; ^3^ Medical Biochemistry Department, Translational and Applied Science Hub(TASH), Faculty of Medicine Ain Shams University, Cairo, Egypt; ^4^ Molecular Biology Lab, Faculty of Oral and Dental Medicine, Misr International University Cairo, Cairo, Egypt; ^5^ Biological Prevention department, Ministry of Defense, Cairo, Egypt

**Keywords:** angiogenesis, bioinformatics, biomarker, ischemic heart disease, RNA panel, type 2 diabetes

## Abstract

**Introduction:**

Diabetes mellitus is a major independent determinant of cardiovascular morbidity. Therefore, we evaluated whether a molecular RNA panel comprising *FZD5* and *GTF2I* could facilitate the early detection and discrimination of ischemic heart disease in individuals with type 2 diabetes mellitus.

**Methods:**

We implemented a two-stage bioinformatics workflow to identify and validate two mRNA candidates associated with T2DM and IHD. Subsequently, we delineated non-coding RNAs linked to these transcripts and the pathways potentially implicated in T2DM complicated by IHD. Finally, we conducted a pilot case–control study and quantified the panel members by RT-qPCR in 56 patients with T2DM, 25 with IHD, 26 with combined T2DM+IHD, and 60 matched controls.

**Results:**

Differential expression analysis showed upregulation of hsa-miR-1976, *FZD5*, and *GTF2I*, accompanied by downregulation of *LINC02210* in the T2DM+IHD group versus controls. The RNA panel achieved high discriminatory performance (AUC = 0.94) between T2DM+IHD and controls, highlighting its potential as a discriminatory tool.

**Discussion:**

this study identified clinically relevant non-coding RNA–based angiogenesis panel (*FZD5*, *GTF2I* mRNAs, hsa-miR-1976 and *LINC02210* lncRNA) as a biomarker signature associated with type 2 diabetes mellitus complicated by ischemic heart disease.

## 1 Introduction

Diabetes mellitus (DM) is a chronic metabolic disorder characterized by sustained hyperglycemia resulting from inadequate insulin secretion and/or impaired insulin sensitivity. Its global burden has risen markedly, establishing DM as a major public health concern (1). Without effective control, DM progressively affects multiple organ systems, with prominent involvement the peripheral nerves, vasculature, and cardiovascular system (2). Within the Middle East and North Africa region, the International Diabetes Federation reports that Egypt carries a substantial diabetes burden: approximately 13.2 million adults are currently affected, with projections reaching 24.7 million by 2050. Egypt is also ranks among the top countries worldwide in both adult prevalence and the absolute number of affected individuals aged 20–79 years (3).

Diabetes mellitus is classified according to underlying the etiological mechanisms that culminate in hyperglycemia. Type 1 diabetes results from immune-mediated destruction of pancreatic β-cells and, although commonly manifesting in childhood or adolescence, may occur at any age; lifelong insulin replacement is required. Type 2 diabetes, the predominant form, arises from insulin resistance with and/or impaired secretion and is strongly associated with obesity; its occurrence in younger age groups has increased in parallel with the global rise in obesity rates. Gestational diabetes mellitus (GDM) is diagnosed during pregnancy and typically resolves after delivery; nevertheless, it confers a substantial long-term risk of developing type 2 diabetes for both the mother and offspring (4).

Prediabetes represents an intermediate state of impaired glucose regulation preceding overt type 2 diabetes, in which glucose values exceed physiological norms but remain below discriminatory thresholds (5).This stage is typically characterized by early β-cell dysfunction and insulin resistance, and accumulating evidence indicates that subclinical complications including neuropathy, nephropathy, retinopathy, and macrovascular alterations may emerge during this stage (6).

In clinical endocrinology, the primary goals are to achieve and maintain optimal glycemic control and to prevent the onset and progression of diabetes-related complications. Accordingly, elucidating the molecular basis of type 2 diabetes is essential for precise target identification and for the rational development and evaluation of mechanism-based precision therapies (7).

Diabetes mellitus is an independent determinant of cardiovascular risk across a broad spectrum of conditions, including cerebrovascular disease, coronary artery disease, and peripheral arterial disease and this burden justifies integrating structured cardiovascular risk stratification within routine diabetes care (8). Patients with diabetes exhibit a markedly increased susceptibility to both macrovascular and microvascular pathologies compared with non-diabetic individuals. In this context, precision medicine has emerged as a transformative paradigm, that enables the tailoring of therapeutic strategies to individual patient profiles with the goal of reducing the incidence and severity of major diabetic complications such as cardiovascular dysfunction, retinopathy, nephropathy, neuropathy, and premature mortality (9).

While lifestyle modification remains foundational, pharmacotherapy is pivotal for controlling hyperglycemia, supporting hepatic function, and mitigating cardiovascular risk (10).

Ischemia results from compromised oxygen supply, diminished nutrient delivery, and impaired clearance of metabolic byproducts. Notably, ischemic manifestations-particularly ischemic heart disease, may precede the formal diagnosis of diabetes mellitus (11). These observations underscore the need for candidate noninvasive biomarkers that enable earlier recognition and refined risk stratification. In routine care, biomarkers support screening, diagnosis, and longitudinal monitoring, and inform the selection of targeted molecular therapies as well as the evaluation of therapeutic response (12).

Insulin resistance is a core lesion in T2DM and denotes attenuated cellular responsiveness to insulin. At the molecular level, defects at canonical signaling nodes—including insulin receptor substrate (IRS) proteins and the PI3K/Akt cascade—are key contributors. Persistent, low-grade inflammation driven by cytokines such as IL-6 and TNF-α disrupts insulin signaling, while mitochondrial dysfunction reduces ATP production and heightens oxidative stress, thereby aggravating resistance. Endoplasmic reticulum stress further impairs insulin action by perturbing protein folding and activating stress-response programs (13). In parallel, epigenetic processes such as DNA methylation and histone modifications reprogram gene-expression profiles that govern insulin sensitivity and β-cell function. Together, these mechanisms illustrate the multifactorial basis of T2DM, integrating genetic susceptibility with environmental and lifestyle factors. Delineating these pathways supports the development of precision-oriented preventive and therapeutic strategies (14).

Disruption of epigenetic regulation is increasingly recognized as a key driver of insulin resistance and the pathogenesis of T2DM. Aberrant epigenetic modifications, often induced by environmental exposures such as dietary patterns and lifestyle behaviors, can remodel chromatin architecture, thereby influencing the accessibility of the transcriptional machinery to target gene loci. These changes may perturb the expression of genes essential for maintaining metabolic homeostasis and insulin sensitivity (15).

MicroRNAs are small, single-stranded non-coding RNAs expressed broadly across tissues. Beyond their canonical role in post-transcriptional repression, some miRNAs can, in defined contexts, enhance gene expression, underscoring their versatile contributions to epigenetic regulation (16). Stable, circulating miRNAs detectable in biofluids have therefore emerged as noninvasive indicators of disease; serum miRNA signatures can mirror tissue-specific pathobiology (17). Recent investigations have demonstrated the utility of miRNA-based assays for the early detection of ischemic heart disease (IHD), highlighting their translational potential in cardiovascular discrimination (18).

Long non-coding RNAs (lncRNAs) are transcripts >200 nucleotides that lack protein-coding capacity. Through interactions with DNA, RNA, and proteins, lncRNAs regulate gene expression at multiple levels spanning epigenetic remodeling, transcriptional control, post-transcriptional processing, and translation (19). At the level of transcription, lncRNAs participate in chromatin reorganization and histone modification, thereby influencing the coordinated activation or repression of defined gene programs. An expanding body of evidence identifies lncRNAs as important epigenetic regulators in the pathogenesis of diabetes and its vascular and metabolic complications. Their contributions to glucose homeostasis and to trajectories of disease progression underscore their promise as discriminatory biomarkers and as candidate therapeutic targets in the management of diabetes (20).

In this study, we applied bioinformatics analyses to delineate the elevated expression of *FZD5*, hsa-miR-1976 and CRHR1-IT1 associated with type 2 diabetes mellitus complicated by ischemic heart disease, and evaluated whether their serum abundances could serve as noninvasive biomarker panel for early detection.

## 2 Results

### 2.1 Bioinformatics results

Differentially expressed genes (DEGs). After standard preprocessing and normalization of the microarray datasets, we identified DEGs in both GSE30122 and GSE19339 using predefined thresholds. In GSE30122, a total of 4, 567 DEGs were detected when comparing of diabetic kidney samples with healthy control kidney samples, including 2, 404 upregulated and 2, 163 downregulated genes (**Supplementary Figure S1A**). In GSE19339, comparing thrombus leukocytes from acute coronary syndrome (ACS) samples (n = 4) with peripheral blood leukocytes from healthy controls (n = 4) yielded 5, 985 DEGs, comprising 2, 309 significantly upregulated and 3, 676 downregulated genes (**Supplementary Figure S1B**). When DEGs from GSE30122 and GSE19339 were intersected in a Venn diagram, 1, 683 common genes were identified (**Supplementary Figure S1C**).

A total of 864 enriched Gene Ontology biological process (GO-BP) terms and 139 Reactome pathways were identified. Functional annotations of the common DEGs were enriched mainly in angiogenesis, hypoxia, platelet degranulation, and cell adhesion. The top eight terms for both GO-BP enrichment are presented in **Supplementary Figure S2**, according to the order of p value. In addition, three angiogenesis-related GO-BP terms with high protein percentages were among the most significant results. Consequently, the GO-BP analysis was utilized to retrieve the gene sets related to angiogenesis to investigate its role in progression of both diseases (**Table 1**).

**Table 1:** Angiogenesis related genes.

Biological process	P-value	Percentage of proteins	Gene set
Angiogenesis	1.37E-09	3.3%	CD160, MMP14, VEGFA, NRP1, NRP2, FN1, SRPX2, THY1, SAT1, ANXA2, MMP2, COL4A2, FZD5, HMOX1, ESM1, CXCL8, CCL2, GLUL, FLT1, TYMP, EFNA1, PTPRB, COL8A2, EPHB2, TEK, HEY1, RORA, KDR, COL15A1, MCAM, HIF1A, EPHB4, JAG1, CAV1, TGFBR3, CALD1, CASP8, TGFBI, CYP1B1, NUS1, ROBO4, HIF3A, ARHGAP22, PLXDC1, HOXB13, MYDGF, EPAS1, NAA15, NRXN3, ANGPTL2
Positive regulation of angiogenesis	1.3E-05	1.82%	PRKCB, HSPB6, CD40, FGF2, ITGB1, ITGB8, CYBB, ADM, PAK4, CX3CR1, RUNX1, SPHK1, HIPK2, HGF, RLN2, C3, BTG1, C3AR1, HMGA2,
Negative regulation of angiogenesis	0.000876	1.17%	PGK1, GTF2I, ROCK1, MECP2, KRIT1, TNMD, PML, GPR4, THBS2, CTNNB1, STAB1, PTN, TGFB2, SPARC, HLA-G
Regulation of angiogenesis	0.0184	0.45%	EMP2, NF1, MAPK7, HMOX1, EFNA1

Following the retrieval of angiogenesis-related gene sets, a PPI network was constructed using the STRING tool (**Supplementary Figure S3A**). The network comprised 87 nodes and 1, 198 edges and showed highly significant enrichment (PPI enrichment p < 1.0 × 10^-16^). We then characterized network topology using the centrality indices betweenness, closeness, and degree for the angiogenesis-related genes. Nodes with degree > 5 were designated as hub genes.

*FZD5* and *GTF2I* were selected for targeted co-regulatory network construction and were validated by Comparative Toxicogenomics Database(CTD) (http://ctdbase.org/) and other databases to be involved in angiogenesis and to be implicated in both acute coronary syndrome and diabetic nephropathy progression (**Supplementary Figures S4-S5**). has-miR-1976 was found to interact with the selected genes, *FZD5* and *GTF2I* (**Supplementary Figures S6**) and was strongly linked to acute coronary syndrome and diabetic nephropathy progression (**Supplementary Figure S7**). LncBase predicted version 3 (DIANA Tools - miRNA-lncRNA interactions (uth.gr) was used to predict interactions between LINC02210 (lncRNAs) and the chosen candidate genes (*FZD5, GTF2I*), and Clustal Omega multiple-sequence alignment was applied to verify the interaction between hsa-miR-1976 and LINC02210 (https://www.ebi.ac.uk/jdispatcher/msa/clustalo) see in (**Supplementary Figure S8**). further verification of the lncRNA annotation was performed using Gene card (GeneCards - Human Genes | Gene Database | Gene Search).

### 2.2 Analysis of biochemical and clinical parameters

The cohort comprised 167 participants allocated to four groups: 60 healthy controls; 25 individuals with IHD; 56 patients meeting ADA criteria for T2DM without cardiovascular disease; and 26 patients meeting ADA criteria for T2DM with cardiovascular disease. Age and sex did not differ significantly across groups (*p* ≥ 0.05). By contrast, the groups differed significantly in smoking status and family history of T2DM (*p* < 0.001); in fasting and 2-h postprandial glucose (*p* < 0.001); in HbA1c and fasting insulin (*p* < 0.001); in HOMA-IR, HOMA-B, and BMI (*p* < 0.001); in systolic/diastolic blood pressure, ALT, AST, CK-MB, and troponin (*p* < 0.001); in the lipid profile—total cholesterol, LDL-C, HDL-C, triglycerides (*p* < 0.001); and in the urine albumin to creatinine ratio (*p* < 0.001), as detailed in **Table 2**.

**Table 2:** Clinical and laboratory characteristics among the groups of the study.

Variable		Healthy control	IHD	T2DM without complication	T2DM + IHD	P-value (overall significance)
N		60	25	56	26	–
Gender	Male	22 (36.6%)	9 (36%)	19 (33.9%)	12 (46.2%)	0.760
	Female	38 (63.3%)	16 (64%)	37 (66.1%)	14 (53.8%)	
Smoking	Smoker	8 (13.3%)	16 (64%)	43 (76.8%)	14 (53.8%)	0.00**
	Non-smoker	51 (85%)	7 (28%)	11 (19.6%)	9 (31%)	
	X-smoker	1 (1.7%)	2 (8%)	2 (3.6%)	3 (15.2%)	
Family History	Positive	0 (0%)	22 (88%)	48 (85.7%)	23 (88.5%)	0.00**
	Negative	60 (100%)	3 (12%)	8 (12.3%)	3 (11.5%)	
Age		51.95 ± 0.906	56.2 ± 1.818	53.95 ± 1.134	55.38 ± 1.349	0.08
Duration Of Diabetes		0.000	13.4 ± 1.385	3.48 ± 0.63	13.77± 0.808	0.00**
Fasting Glucose (mg/dL)		90.13 ± 1.82	196.48 ± 15.98	149.68 ± 11.16	41.62 ± 8.16	0.00**
Post Prandial Glucose (mg/dL)		110.12 ± 2.075	297.92 ± 23.68	216.71 ± 11.92	325.19 ± 21.73	0.00**
Glycated haemoglobin HbA1c (%)		3.83 ± 0.159	9.6 ± 0.616	5.37 ± 0.22	9.7 ± 0.64	0.00**
Insulin (IU Per ml)		5.25 ± 0.32	15 ± 0.54	3.677 ± 0.49	16.42 ± 0.616	0.00**
HOMA_IR		0.856 ± 0.078	5.31 ± 0.54	5.048 ± 0.46	6.68 ± 0.57	0.00**
HOMA-B		200.3 ± 2.47	53.08 ± 1.73	97.48 ± 5.63	51.76 ± 1.53	0.00**
Systolic Blood pressure		117.75 ± 1.038	133.6 ± 2.17	137.32 ± 2.24	138.08 ± 2.88	0.00**
Diastolic Blood pressure		76.58 ± 0.667	88.6 ± 1.9	90.8 ± 1.71	90 ± 1.75	0.00**
BMI (kg/m2)		31.16 ± 0.64	34.06 ± 1.34	35.29 ± 0.66	35.34 ± 1.17	0.00**
Total Cholesterol (mg/dL)		100.4 ± 3.18	294.32 ± 14.69	289.88 ± 5.54	327.58 ± 11.93	0.00**
LDLc (mg/dL)		73.97 ± 2.28	199.24 ± 7.58	189.04 ± 4.52	220.04 ± 12.19	0.00**
HDLc (mg/dL)		67.62 ± 1.16	31.04 ± 1.25	39.95 ± 1.62	32.62 ± 1.31	0.00**
TGs (mg/dL)		102.32 ± 1.63	310.36 ± 11.24	238.30 ± 9.08	307.15 ± 8.85	0.00**
Alb_Creat Ratio		13.4 ± 0.423	24.72 ± 0.89	23.55 ± 0.64	26.46 ± 0.78	0.00**
ALT (IU/L)		41.87 ± 1.92	41.32 ± 1.85	51.88 ± 2.27	44.08 ± 2.34	0.00**
AST (IU/L)		38.97 ± 1.72	66.32 ± 12.46	49.23 ± 2.5	51.8 ± 2.28	0.00**
CKMB		7.02 ± 0.64	47.42 ± 4.58	40.04 ± 4.23	40.17 ± 5.00	0.00**
Troponin		0.609 ± 0.104	31.18 ± 4.9	14.73 ± 3.49	50.54 ± 8.31	0.00**

Values represent means ± SEM. ** *p* < 0.01: is highly significant, * *p* < 0.05: is significant, p > 0.05: is not significant.

The results were analyzed by crosstabulation, Pearson chi-square and ANOVA.

### 2.3 Evaluation of circulating mRNA, miRNAs, LncRNA in IHD, T2DM without complications, T2DM complicated with IHD patients compared to healthy subjects

We assessed differential expression of the selected RNA panel across study groups using fold-change analysis. Relative to controls, expression of panel members other than LINC02210 including *FZD5, GTF2I*, and hsa-miR-1976 increased stepwise from controls to T2DM (without complications) and IHD, with the highest levels in T2DM+IHD (*p* < 0.001). By contrast, LINC02210 showed a progressive decrease from controls → T2DM (without complications) → T2DM+IHD, reaching its lowest abundance in IHD (*p* < 0.001). Consistent with these trajectories, *FZD5, GTF2I*, and hsa-miR-1976 were significantly upregulated in IHD, T2DM without complications, and T2DM+IHD versus healthy controls, whereas the overall reduction in LINC02210 across groups did not reach statistical significance (*P* > 0.05), as summarized in **Table 3**.

**Table 3:** Descriptives and one-way ANOVA of RNA panel expression among the study groups.

Groups Gene	Healthy control	IHD	T2DM without complication	T2DM +IHD	P-value (one-way ANOVA)		
					Overall significance	Between-group significance	
FZD5	1± 0.60 1.08	21.20 ± 12.51	3.15 ± 2.25	27.61 ± 20.82	0.00**	^a^ 0.00^**^ ^b^ 0.819 ^c^ 0.00^**^	^d^ 0.00^**^ ^e^ 0.083 ^f^ 0.00^**^
GTF2I	0.707± 2.29	7.72 ± 8.72	5.98 ± 2.73	22.53 ± 26.57	0.00**	^a^ 0.044^*^ ^b^ 0.056 ^c^ 0.00^**^	^d^ 0.914 ^e^ 0.00^**^ ^f^ 0.00^**^
miR-1976	0.953 ± 0.959	565.01 ± 1,446.2	105.19 ± 176.54	679.81 ± 1,378.2	0.00**	^a^ 0.015* ^b^ 0.890 ^c^ 0.00^**^	^d^ 0.072 ^e^ 0.953 ^f^ 0.012^*^
LINC02210	399.93 ± 1,405.43	0.0434 ± 0.0603	14.27 ± 21.87	0.101 ± 0.208	0.04*	^a^ 0.197 ^b^ 0.071 ^c^ 0.187	^d^ 1.0 ^e^ 1.0 ^f^ 1.0

-The relative expression of the selected RNAs axis were evaluated in our study subjects and the differences in fold changes were analyzed by one-way ANOVA test, post Hoc test and Kruskal Wallis test of RNA panel expression among the study groups.

**Figure 1:**
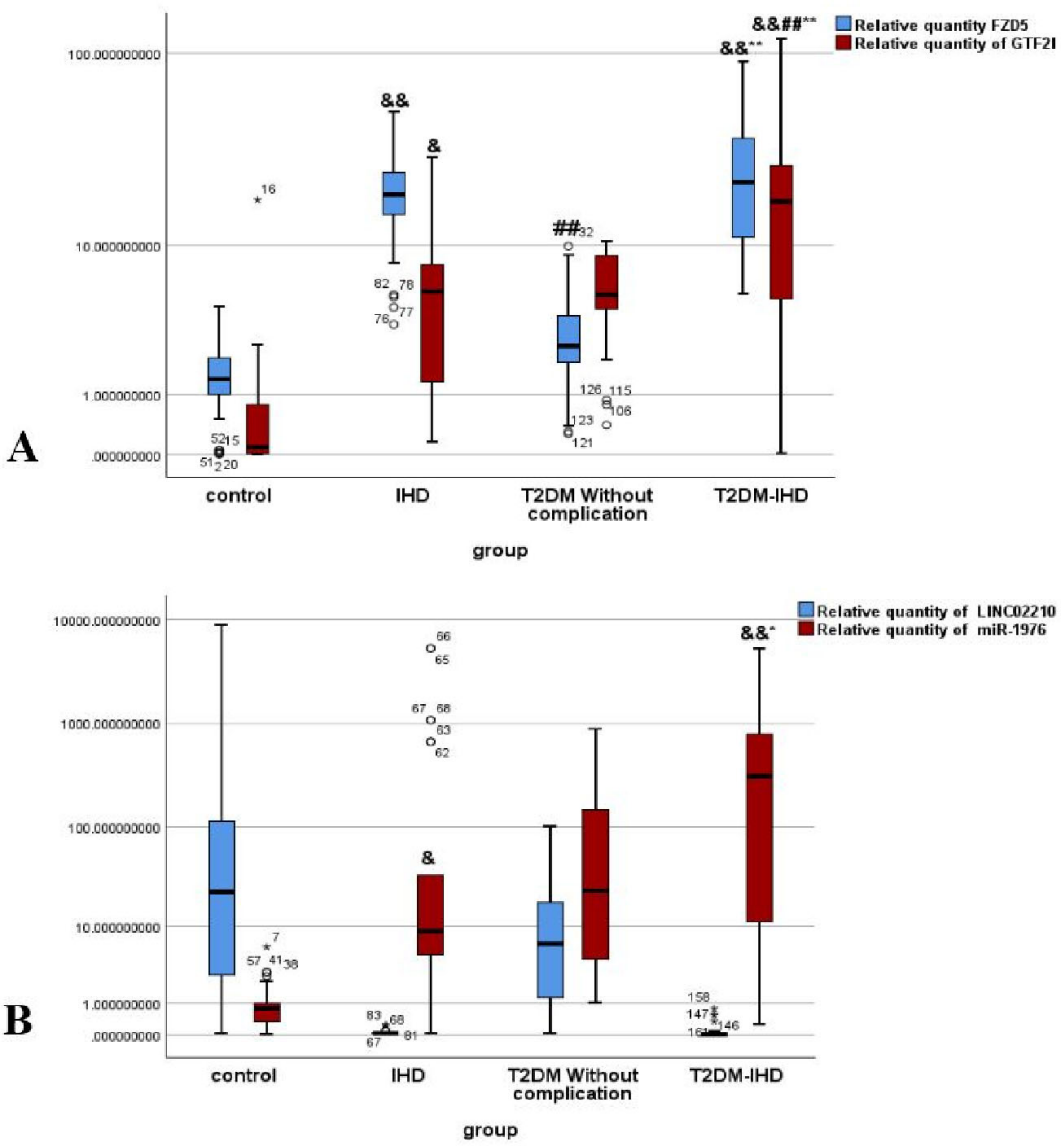
Relative expression of circulatory RNAs panel among the study groups.

-Results presented as Values represent means ± SD, Mean, St. Deviation, Mean ranks, interquartile ranges (IQR). Levels of FZD5 mRNA, GTF2I mRNA, miR-1976, and LINC02210 are depicted in table 3 and figure 1, A&B.

-^a^ Control vs. IHD, ^b^ Control vs. T2DM without Complication, ^c^ Control vs. T2DM +IHD, ^d^ IHD vs. T2DM without Complication, ^e^ IHD vs. T2DM +IHD, ^f^ T2DM without Complication vs. T2DM +IHD. ** p < 0.01; * p < 0.05.

### 2.4 Assessment of plasma biomarkers in obese and diabetic patients relative to healthy controls

Blood-derived biomarkers provide practical tools for monitoring, diagnosis, and disease staging in T2DM+IHD. In this work, we profiled a panel of biomarkers previously implicated in T2DM+IHD progression. Plasma *FZD5* and *GTF2I* markers linked to cardiovascular pathology were significantly elevated in IHD and T2DM+IHD compared with healthy controls (**Figure 1A**). Moreover, the T2DM+IHD group showed further elevations in both markers when relative to controls controls, IHD, and T2DM without complications (**Figure 1A**). Discriminatory performance for separating T2DM from IHD was greater for *FZD5* mRNA than for *GTF2I* mRNA (**Figure 1A**). LINC02210 levels may reflect adipose-tissue dysfunction relevant to the progression of T2DM+IHD and IHD; in compared with healthy subjects, LINC02210 was significantly reduced in both patient groups (**Figure 1B**). Conversely, plasma hsa-miR-1976 concentrations were significantly higher in T2DM+IHD, IHD, and T2DM without complications than in healthy controls (**Figure 1B**).

### 2.5 Discriminatory performance of RNAs panel among the study groups assessed by ROC curve analysis

**Figure 2:**
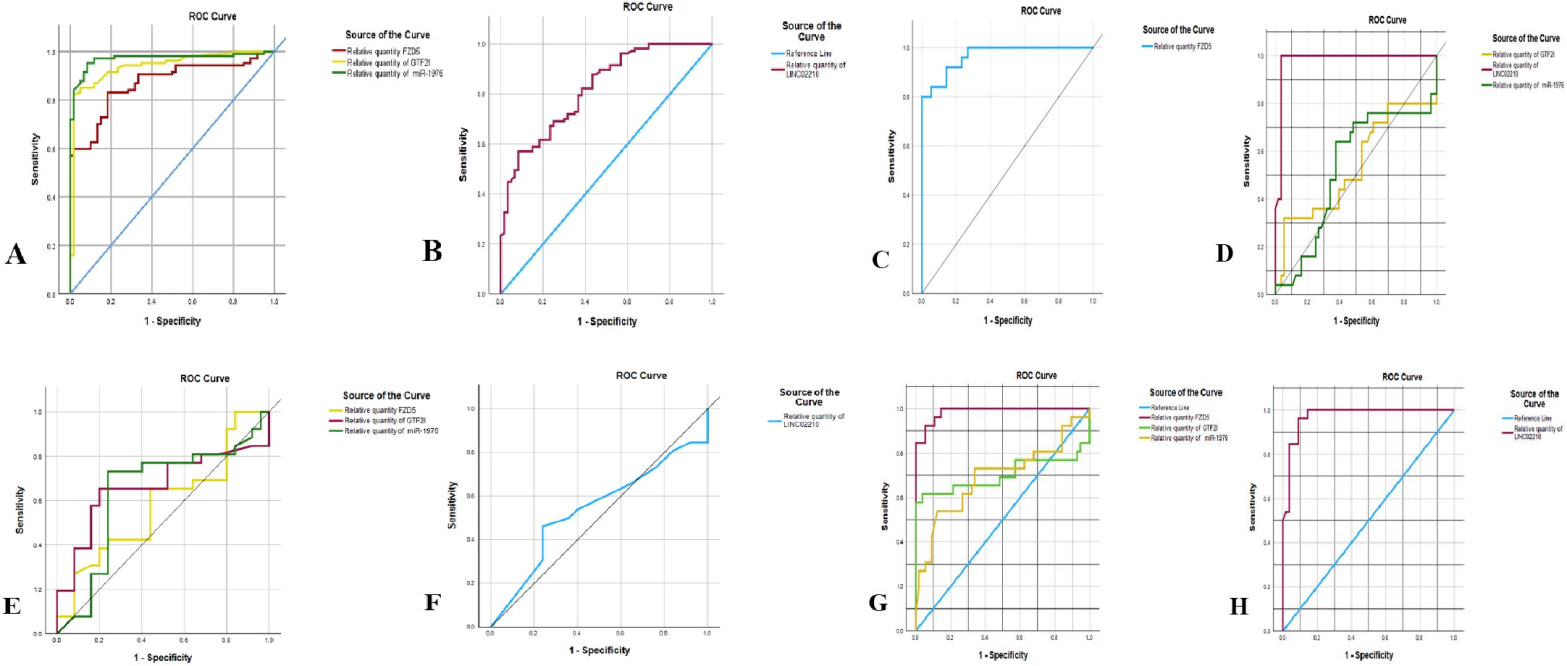
Discriminatory Performance, (ROC Curve Analysis).

We evaluated the discriminatory performance of the dysregulated RNA panel using receiver operating characteristic (ROC) analyses across multiple contrasts: diseased vs controls, IHD vs T2DM, IHD vs T2DM+IHD, and T2DM vs T2DM+IHD. For each individual RNA, we derived optimal cutoff values and computed sensitivity, specificity, positive predictive value (PPV), negative predictive value (NPV), and overall accuracy. Comprehensive performance metrics are provided in **Table 4** and **Figures 2A–H**.

**Table 4:** Discriminatory performance of RNAs panel among the study groups assessed by ROC curve analysis.

T2DM vs. T2DM+IHD											
Biomarker	AUC	SE	P-value	95% CI		Optimal cut-off value	Sensitivity	Specificity	PPV	NPV	Accuracy
				Lower bound	Upper bound						
FZD5 mRNA	0.986	0.009	0.000	0.969	1.004	> 5.8718	96.2%	89.3%	74.19%	94.12%	86.59%
GTF2I mRNA	0.702	0.081	0.013	0.543	0.861	> 5.290	69.2%	51.8%	40%	78.38%	57.32%
hsa-miR-1976 miRNA	0.694	0.070	0.006	0.556	0.832	> 51.4426	73.1%	66.1%	50%	84.09%	68.29%
LINC02210	0.973	0.014	0.000	0.944	1.002	< 0.8189	100%	85.71%	76.47%	100%	90.24%

*p* < 0.01: is highly significant, p < 0.05: is significant, p > 0.05: is not significant.

AUC, area under the curve; SE, stander error of mean, PPV, positive predictive value and NPV: negative predictive value.

#### 2.5.1 Diseased groups *versus* controls

Against healthy participants, discriminatory performance yielded AUCs of 0.870 (*FZD5* mRNA), 0.940 (*GTF2I* mRNA), 0.970 (hsa-miR-1976), and 0.819 (LINC02210). The corresponding optimal cutoff values were 1.732, 0.960, 1.774, and 9.10013 for *FZD5, GTF2I*, hsa-miR-1976, and LINC02210, respectively. Estimated sensitivities were 90.7%, 91.6%, 97.2%, and 82.2%, with specificities of 66.7%, 81.7%, 88.3%, and 61.7%. Collectively, these metrics indicate that the RNA panel can separate patient groups from controls (**Table 4**, **Figures 2A, B**).

#### 2.5.2 IHD group versus T2DM

In the IHD vs T2DM comparison, *FZD5* mRNA (AUC 0.966) and LINC02210 (AUC 0.978) achieved clear discrimination. The corresponding optimal cutoff values were 4.2156 and 0.2753, yielding sensitivities of 96.0% and 100% and specificities of 76.8% and 96.4%, respectively. By contrast, *GTF2I* mRNA and hsa-miR-1976 did not discriminate between IHD from T2DM, reflecting lower AUCs and suboptimal operating characteristics (**Table 4**, **Figures 2C, D**).

#### 2.5.3 IHD versus T2DM+IHD

The results represent the candidate RNAs panel that did not effectively discriminate IHD cases from T2DM+IHD (**Table 4**, **Figures 2E, F**).

#### 2.5.4 T2DM versus T2DM+IHD

We next appraised the performance of the mRNA/miRNA/lncRNA panel for distinguishing T2DM from T2DM+IHD using ROC analysis. The optimal cutoff values were 5.8718 *(FZD5* mRNA), 5.290 (*GTF2I* mRNA), 51.4426 (hsa-miR-1976), and 0.8189 (LINC02210). The corresponding AUCs were 0.986, 0.702, 0.694, and 0.973, respectively. Estimated sensitivities reached 96.2%, 69.2%, 73.1%, and 100%, with specificities of 89.3%, 51.8%, 66.1%, and 85.71%. These findings are concordant with the bioinformatics signal and indicate that the proposed RNA panel may aid discriminatory separation of T2DM+IHD from T2DM (see **Table 4**, **Figures 2G, H**).

### 2.6 Correlation between biomarker positivity rate and clinicopathological factors in disease groups

Among positive values of *FZD5* mRNA, *GTF2I* mRNA, has-miR-1976 miRNA, LINC02210 LncRNA and various clinicopathological factors across different disease groups, our analysis revealed that Hemoglobin A1c (HbA1c, %), Total Cholesterol (mg/dL), triglycerides(mg/dL), CK-MB and Troponin have a significant positive correlation with RNA panel among all diseased groups. On the other hand, sex, ALT(IU/L), BMI (kg/m²), and Age showed no significant correlation with RNA panel among diseased groups (**Table 5**).

**Table 5:** Correlation between biomarkers positivity rate and clinicopathological factors in diseased groups.

Disease vs. Control											
Biomarker	AUC	SE	*p*-value	95% CI		Optimal cut-off value	Sensitivity	Specificity	PPV	NPV	Accuracy
				Lower bound	Upper bound						
FZD5 mRNA	0.870	0.027	0.000	0.817	0.924	> 1.732	90.7%	66.7%	82.9%	80%	82%
GTF2I mRNA	0.940	0.020	0.000	0.901	0.979	> 0.96	91.6%	81.7%	90%	86%	88.6%
hsa-miR-1976 miRNA	0.970	0.013	0.000	0.945	0.996	> 1.774	97.2%	88.3%	93.7%	94.6%	94%
LINC02210	0.819	0.033	0.000	0.755	0.883	< 9.10013	82.2%	61.7%	20.7%	66.1%	74.9%
IHD vs. T2DM											
Biomarker	AUC	SE	*p*-value	95% CI		Optimal cut-off value	Sensitivity	Specificity	PPV	NPV	Accuracy
				Lower bound	Upper bound						
FZD5 mRNA	0.966	0.018	0.000	0.932	1.001	> 4.2156	96%	76.8%	64.86%	97.73%	82.72%
GTF2I mRNA	0.539	0.077	0.612	0.388	0.689	< 6.1850	64%	42.9%	33.33%	72.73%	49.38%
hsa-miR-1976 miRNA	0.529	0.074	0.695	0.384	0.673	< .9755	72%	51.8%	40%	80.56%	58.02%
LINC02210	0.978	0.016	0.000	0.947	1.009	< 0.2753	100%	96.4%	92.59%	100%	97.53%
IHD vs. T2DM+IHD											
Biomarker	AUC	SE	*p*-value	95% CI		Optimal cut-off value	Sensitivity	Specificity	PPV	NPV	Accuracy
				Lower bound	Upper bound						
FZD5 mRNA	0.580	0.081	0.325	0.421	0.739	> 19.7510	65.4%	56%	60.71%	60.87%	60.78%
GTF2I mRNA	0.666	0.080	0.038	0.509	0.823	> 8.40	65.4%	80%	77.27%	68.97%	72.55%
hsa-miR-1976 miRNA	0.640	0.083	0.092	0.477	0.803	> 42.9228	73.1%	76%	76%	73.08%	74.51%
LINC02210	0.537	0.083	0.655	0.375	0.699	< 0.025	53.8%	60%	44.44%	41.67%	43.14%

**Table:** 

IHD vs. T2DM													
Gene Variable		FZD5 mRNA			GTF2I mRNA			has-miR-1976			LINC02210		
		IHD	T2DM	*p-value*	IHD	T2DM	*p-value*	IHD	T2DM	*p-value*	IHD	T2DM	*p-value*
Gender	Male	24 (37.5 %)	38 (36.9%)	0.937	47 (40.5%)	15 (29.4%)	0.171	41 (36.6%)	21 (38.2%)	0.843	22 (42.3%)	40 (34.8%)	0.351
	Female	40 (62.5 %)	65 (63.1 %)		69 (59.5%)	36 (70.6%)		71 (63.4%)	34 (61.8%)		30 (57.7%)	75 (65.2%)	
Smoking	Smoker	39 (60.9%)	42 (40.8%)	0.00**	45 (38.8%)	36 (70.6%)	0.00**	46 (41.4%)	35 (63.6%)	0.00**	29 (55.8%)	52 (45.2%)	0.032
	Non-smoker	20 (31.3%)	58 (56.3%)		63 (54.3%)	15 (29.4%)		64 (56.1%)	14 (25.5%)		18 (34.6%)	60 (52.2%)	
	X-smoker	5 (7.8 %)	3 (2.9%)		8 (6.9%)	0 (0%)		2 (1.8%)	6 (10.9%)		5 (9.6%)	3 (2.6%)	
Family History	Positive	55 (85.9%)	38 (36.9%)	0.00**	52 (44.8%)	41 (80.4%)	0.00**	47 (42%)	46 (83.6%)	0.00**	45 (86.5%)	48 (41.7%)	0.00**
	Negative	9 (14.1%)	65 (63.1%)		64 (55.2%)	10 (19.6%)		65 (58%)	9 (16.4%)		7 (13.5%)	67 (58.3%)	
Age		56.05 ± 0.991	52.39 ± 0.753	0.00**	53.29 ±0.77	54.92 ± 0.99	0.223	53.60 ± 0.765	54.18 ± 1.031	0.565	55.44 ± 1.061	53.04 ± 0.744	0.07
Duration Of Diabetes		12.078 ± 0.7796	1.117 ± 0.3076	0.00**	3.897 ± 0.63	8.55 ±0.91	0.00**	3.353 ± 0.652	9.318 ± 0.732	0.00**	12.31 ± 0.896	2.157 ± 0.425	0.00**
Fasting Glucose (mg/dL)		195.23 ± 9.031	108.36 ± 5.028	0.00**	125.95 ± 6.004	177.37 ± 11.16	0.00**	111.96 ± 3.813	202.13 ± 11.774	0.00**	191.9 ± 9.799	118.93 ± 5.84	0.00**
Post Prandial Glucose (mg/dL)		302.86 ± 14.488	148.18 ± 6.502	0.00**	172.73 ± 8.20	286.45 ± 18.56	0.00**	165.38 ± 8.546	293.16 ± 15.62	0.00**	307.96 ± 17.163	162.02 ± 7.26	0.00**
Glycated hemoglobin HbA1c (%)		8.816 ± 0.416	4.46 ± 0.162	0.00**	5.56 ± 0.285	7.41 ± 0.45	0.00**	5.22 ± 0.25	7.98 ± 0.48	0.00**	9.32 ± 0.474	4.69 ± 0.166	0.00**
Insulin (IU Per ml)		15.50 ± 0.418	9.79 ± 0.602	0.00**	10.2 ± 0.548	16.02 ± 0.479	0.00**	10.25 ± 0.58	15.49 ± 0.451	0.00**	14.87 ± 0.542	10.67 ± 0.578	0.00**
HOMA_IR		5.71 ± 0.363	2.67± 0.317	0.00**	3.09 ± 0.31	5.51 ± 0.428	0.00**	2.78 ± 0.269	5.98 ± 0.477	0.00**	5.64 ± 0.423	3.02 ± 0.306	0.00**
HOMA-B		57.70± 2.841	159.78 ± 5.579	0.00**	142.25 ± 6.353	71.55 ± 4.66	0.00**	148.52 ± 6.188	63.93 ± 3.226	0.00**	60.67 ± 4.785	147.78 ± 5.783	0.00**
Systolic Blood pressure		135.78 ± 1.726	126.17 ± 1.570	0.00**	126.51 ± 1.39	137.45 ± 2.12	0.00^**^	126.56 ± 1.425	136.55 ± 2.063	0.00**	133.94 ± 1.733	128 ± 1.571	0.012*
Diastolic Blood pressure		89.53 ± 1.213	82.57 ± 1.158	0.00**	83.45 ± 1.07	89.31 ± 1.46	0.00**	82.59 ± 1.044	90.64 ± 1.422	0.00**	88.46 ± 1.335	83.78 ± 1.119	0.014^*^
BMI (kg/m2)		34.87 ± 0.759	32.87 ± 0.526	0.027*	33.14 ± 0.54	34.75 ± 0.75	0.092	33.02 ± 0.55	34.87 ± 0.70	0.049*	34.91 ± 0.853	33.06 ± 0.5	0.052
Total Cholesterol (mg/dL)		308.28 ± 8.601	178.66 ± 9.508	0.00**	196.52 ± 9.96	300.71 ± 8.94	0.00**	189.2 ± 9.81	308.04 ± 8.08	0.00**	296.92 ± 11.48	197.32 ± 9.593	0.00**
LDLc (mg/dL)		207.77 ± 6.751	120.67 ± 5.748	0.00**	131.34 ± 6.09	205.69 ± 7.42	0.00**	128.15 ± 6.048	206.78 ± 7.048	0.00**	204.54 ± 8.215	131.22 ± 5.916	0.00**
HDLc (mg/dL)		33.84 ± 1.094	55.84 ± 1.718	0.00**	52.61 ± 1.74	35.59 ± 1.30	0.00**	53.31 ± 1.775	35.40 ± 1.155	0.00**	34.27 ± 1.402	53.36 ± 1.673	0.00**
TGs (mg/dL)		300.97 ± 7.300	277.99 ±6.56	0.00**	181.53 ± 9.007	277.86 ± 9.22	0.00**	174.56 ± 8.996	285.05 ± 7.594	0.00**	293.17 ± 10.238	173.77 ± 8.001	0.00**
Alb/Creat Ratio		25.52 ± 0.513	17.44 ± 0.622	0.00**	18.75 ± 0.64	24.59 ± 0.65	0.00**	18.38 ± 0.643	24.93 ± 0.576	0.00**	24.29 ± 0.673	18.83 ± 0.644	0.00**
ALT (IU/L)		43.66 ± 1.444	46.62 ± 1.669	0.181	44.73 ± 1.39	47.20 ±2.18	0.334	45.97 ± 1.512	44.49 ± 1.788	0.554	44.19 ± 1.644	46.07 ± 1.531	0.405
AST (IU/L)		58.44 ± 5.015	42.33 ± 1.647	0.00**	46.95 ± 3.09	52.04 ± 2.15	0.298	46.32 ± 3.214	52.95 ± 1.849	0.167	58.87 ± 6.155	43.82 ± 1.552	0.00**
CKMB		44.47 ± 3.80	19.87 ± 2.20	0.00**	21.27 ± 1.95	47.57 ± 4.77	0.00^**^	20.38 ± 1.93	47.46 ± 4.48	0.00**	40.21 ± 3.25	24.37 ± 2.71	0.00**
Troponin		36.31 ± 4.55	6.12 ± 1.64	0.00**	13.68 ± 2.59	26.82 ± 4.52	0.01*	8.83 ± 1.99	35.74 ± 4.91	0.00**	36.31 ± 4.89	9.28 ± 2.10	0.00**
IHD vs. T2DM+IHD													
Gene Variable		FZD5 mRNA			GTF2I mRNA			has-miR-1976			LINC02210		
		IHD	T2DM+ IHD	*p-value*	IHD	T2DM+ IHD	*p-value*	IHD	T2DM+ IHD	*p-value*	IHD	T2DM+ IHD	*p-value*
Gender	Male	50 (36.0 %)	12 (42.9%)	0.491	51 (39.8%)	11 (28.2%)	0.188	43 (35.8%)	19 (40.4%)	0.581	9 (36.0%)	53 (37.3%)	0.899
	Female	89 (64 %)	16 (57.1%)		77 (60.2%)	28 (71.8%)		77 (64.2%)	28 (59.6%)		16 (64.0%)	89 (62.7%)	
Smoking	Smoker	64 (46.0%)	17 (60.7%)	0.05*	53 (41.4%)	28 (71.8%)	0.00**	51 (42.5%)	30 (63.8%)	0.00**	16 (64.0%)	65 (45.8%)	0.00**
	Non-smoker	70 (50.4%)	8 (28.6%)		67 (52.3%)	11 (28.2%)		67 (55.8%)	11 (23.4%)		5 (20.0%)	73 (51.4%)	
	X-smoker	5 (3.6 %)	3 (10.7%)		8 (6.3%)	0 (0%)		2 (1.7%)	6 (12.8%)		4 (16.0%)	4 (2.8%)	
Family History	Positive	68 (48.9%)	25 (89.3%)	0.00**	63 (49.2%)	30 (76.9%)	0.00**	51 (42.5%)	42 (89.4%)	0.00**	23 (92.0%)	70 (49.3%)	0.00**
	Negative	71 (51.1%)	3 (10.7%)		65 (50.8%)	9 (23.1%)		69 (57.5%)	5 (10.6%)		2 (8.0%)	72 (50.7%)	
Age		52.97± 0.631	57.86± 1.726	0.00**	53.31 ± 0.71	55.36 ± 1.2	0.159	53.39 ± 0.730	54.81 ± 1.132	0.301	53.84 ± 1.249	53.78 ± 0.689	0.973
Duration Of Diabetes		3.41 ± 0.463	14.79 ± 1.185	0.00**	4.242 ± 0.602	8.846 ± 1.059	0.00**	3.396 ± 0.618	10.22 ± 0.73	0.00**	12.04 ± 0.878	4.134 ± 0.565	0.00**
Fasting Glucose(mg/dL)		129.78 ± 6.015	200.57± 10.38	0.00**	134.79 ± 6.741	164.18 ± 9.29	0.02*	115.46 ± 4.239	208.53 ± 12.6	0.00**	188.68 ± 9.09	133.37 ± 6.233	0.00**
Post Prandial Glucose (mg/dL)		187.84 ± 9.121	304.86 ± 20.58	0.00**	186.09 ± 9.258	277.59 ± 19.975	0.00**	171.03 ± 8.562	300.47± 16.966	0.00**	342.12 ± 24.844	183.75 ± 8.155	0.00**
Glycated hemoglobin HbA1c (%)		5.29 ± 0.213	10.28 ± 0.60	0.00**	5.69 ± 0.27	7.59 ± 0.54	0.00**	5.3 ± 0.239	8.22 ± 0.535	0.00**	10.52 ± 0.585	5.35 ± 0.218	0.00**
Insulin (IU Per ml)		11.16 ± 0.510	16.04 ± 0.569	0.00**	10.6 ± 0.513	16.49 ± 0.573	0.00**	10.56 ± 0.554	15.6 ± 0.507	0.00**	16.28 ± 0.567	11.22 ± 0.503	0.00**
HOMA_IR		3.55 ± 0.296	5.27 ± 0.52	0.015*	3.37 ± 0.31	5.35 ± 0.46	0.00**	2.95 ± 0.265	6.09 ± 0.534	0.00**	6.18 ± 0.576	3.42 ± 0.282	0.00**
HOMA-B		134.60 ± 5.63	51.46 ± 1.519	0.00**	135.66 ± 6.069	71.41 ± 5.733	0.00**	144.28 ± 6.012	60.36 ± 2.79	0.00**	52.0 ± 1.547	132.75 ± 5.613	0.00**
Systolic Blood pressure		128.71± 1.345	135.54 ± 2.762	0.03*	127.5 ± 1.37	137.56 ± 2.337	0.00^**^	127.21 ± 1.368	136.6 ± 2.342	0.00**	135.0 ± 2.345	128.94 ± 1.368	0.078
Diastolic Blood pressure		84.78 ± 1.007	87.50 ± 1.754	0.255	83.91± 1.051	89.62 ± 1.427	0.00**	83.13 ± 1.003	90.64 ± 1.621	0.00**	89.4 ± 1.965	84.51 ± 0.977	0.049^*^
BMI (kg/m2)		33.42 ± 0.478	34.69 ± 1.13	0.283	33.31 ± 0.517	34.69 ± 0.816	0.184	33.07 ± 0.519	35.08 ± 0.80	0.04*	35.2 ± 1.23	33.35 ± 0.469	0.135
Total Cholesterol (mg/dL)		209.75 ± 8.89	320.61 ± 11.999	0.00**	207.16 ± 9.628	297.82 ± 10.341	0.00**	194.29 ± 9.349	315.26 ± 8.84	0.00**	314.48 ± 13.198	213.17 ± 8.907	0.00**
LDLc (mg/dL)		144.73 ± 5.954	200.32 ± 10.18	0.00**	139.47 ± 6.055	201.9 ± 8.914	0.00**	130.89 ± 5.746	213.17 ± 7.761	0.00**	212.32 ± 10.124	143.79 ± 5.780	0.00**
HDLc (mg/dL)		50.45 ± 1.545	32.32 ± 1.416	0.00**	50.79 ± 1.673	36.33 ± 1.473	0.00**	52.38 ± 1.696	34.74 ± 1.248	0.00**	33.44 ± 1.437	49.87 ± 1.548	0.00**
TGs (mg/dL)		190.76 ± 8.047	311.21 ± 7.911	0.00**	191.3 ± 8.771	275.44 ± 10.567	0.00**	176.89 ± 8.508	297.91 ± 6.777	0.00**	300.08 ± 11.276	195.26 ± 8.113	0.00**
Alb/Creat Ratio		19.34 ± 0.563	26.46 ± 0.765	0.00**	19.2 ± 0.599	24.92 ± 0.77	0.00**	18.68 ± 0.621	25.26 ± 0.581	0.00**	25.12 ± 0.771	19.73 ± 0.580	0.00**
ALT (IU/L)		46.49 ± 1.336	40.5 ± 1.981	0.01*	45.43 ± 1.352	45.67 ± 2.366	0.932	46.14 ± 1.49	43.81 ± 1.686	0.302	45.52 ± 2.267	45.48 ± 1.321	0.99
AST (IU/L)		48.60 ± 2.686	48.0 ± 1.528	0.845	46.96 ± 2.814	53.56 ± 2.618	0.215	47.02 ± 3.032	52.3 ± 1.914	0.143	53.32 ± 2.551	47.65 ± 2.602	0.37
CKMB		27.43 ± 2.44	38.58 ± 4.52	0.05*	24.36 ± 2.2	45.52 ± 5.23	0.00^**^	22.52 ± 2.015	46.61 ± 5.067	0.00**	37.48 ± 3.79	27.86 ± 2.47	0.03*
Troponin		14.96 ± 2.43	31.28± 6.15	0.00**	13.68 ± 2.38	30.84 ± 5.59	0.00**	9.51 ± 1.94	38.58 ± 5.49	0.00**	44.4 ± 7.33	12.99 ± 2.17	0.00**

Descriptive statistics, cross tab analysis: Values represent means ± SEM. **p < 0.01, *p < 0.05.

### 2.7 Correlation analysis and linear regression analysis

We examined associations within the RNA panel across study groups using Spearman’s rank correlation. Positive correlations were observed between *FZD5* and *GTF2I* (r = 0.462; *p* < 0.001), between *FZD5* and hsa-miR-1976 (r = 0.632; *p* < 0.001), and between *GTF2I* and hsa-miR-1976 (r = 0.545; *p* < 0.001). In contrast, LINC02210 correlated negatively with *FZD5* (r = −0.651; *p* < 0.001), *GTF2I* (r = −0.369; *p* < 0.001), and hsa-miR-1976 (r = −0.456; *p* < 0.001). Overall, these data indicate significant interrelationships within the RNA network across the analyzed cohorts (**Table 6**).

**Table 6:** Correlation analysis in all groups.

T2DM vs. T2DM+IHD													
Gene Variable		FZD5 mRNA			GTF2I mRNA			has-miR-1976			LINC02210		
		T2DM	T2DM+ IHD	*p-value*	T2DM	T2DM+ IHD	*p-value*	T2DM	T2DM+ IHD	*p-value*	T2DM	T2DM+ IHD	*p-value*
Gender	Male	40 (34.5%)	22 (43.1%)	0.286	43 (39.8%)	19 (32.2%)	0.33	44 (35.8%)	18 (40.9%)	0.545	38 (36.9%)	24 (37.5%)	0.937
	Female	76 (65.5%)	29 (56.9%)		65 (60.2%)	40 (67.8%)		79 (64.2%)	26 (59.1%)		65 (63.1%)	40 (62.5%)	
Smoking	Smoker	54 (46.6%)	27 (52.9%)	0.618	40 (37.0%)	41 (69.5%)	0.00**	53 (43.1%)	28 (63.6%)	0.00**	43 (41.7%)	38 (59.4%)	0.01*
	Non-smoker	57 (49.1%)	21 (41.2%)		60 (55.6%)	18 (30.5%)		68 (55.3%)	10 (22.7%)		57 (55.3%)	21 (32.8%)	
	X-smoker	5 (4.3%)	3 (5.9%)		8 (7.4%)	0 (0%)		2 (1.6%)	6 (13.6%)		3 (2.9%)	5 (7.8%)	
Family History	Positive	49 (42.2%)	44 (86.3%)	0.00**	44 (40.7%)	49 (83.1%)	0.00**	54 (43.9%)	39 (88.6%)	0.00**	44 (42.7%)	49 (76.6%)	0.00**
	Negative	67 (57.8%)	7 (13.7%)		64 (59.3%)	10 (16.9%)		69 (56.1%)	5 (11.4%)		59 (57.3%)	15 (23.4%)	
Age		53.24 ± 0.730	55.04 ± 1.125	0.178	52.56 ± 0.741	56.05± 1.031	0.00**	53.43 ± 0.716	54.80 ± 1.193	0.33	52.76 ± 0.774	55.45 ± 0.981	0.032*
Duration Of Diabetes		2.362 ± 0.428	12.039 ± 0.9750	0.00**	3.037 ± 0.4895	9.492 ± 1.0578	0.00**	3.533 ± 0.613	10.307 ± 0.749	0.00**	1.495 ± 0.35	11.469 ± 0.859	0.00**
Fasting Glucose (mg/dL)		122.28 ± 6.239	185.73 ± 9.507	0.00**	126.22 ± 6.424	169.90 ± 10.01	0.00**	116.97± 4.251	210.66± 13.341	0.00**	114.39 ± 5.761	185.53 ± 9.236	0.00**
Post Prandial Glucose (mg/dL)		174.49 ± 9.628	282.45 ± 15.15	0.00**	170.69± 8.74	274.78 ± 16.563	0.00**	172.52 ± 8.398	305.14 ± 17.91	0.00**	154.9 ± 7.114	292.05 ± 15.436	0.00**
Glycated hemoglobin HbA1c (%)		4.78 ± 0.182	9.20 ± 0.479	0.00**	5.38 ± 0.28	7.5 ± 0.43	0.00**	5.31 ± 0.234	8.44 ± 0.56	0.00**	4.48 ± 0.15	8.79 ± 0.432	0.00**
Insulin (IU Per ml)		10.46 ± 0.572	15.43 ± 0.465	0.00**	9.93 ± 0.575	15.73 ± 0.450	0.00**	10.63 ± 0.545	15.75 ± 0.508	0.00**	10.27 ± 0.604	14.72 ± 0.543	0.00**
HOMA_IR		2.98 ± 0.301	5.77 ± 0.426	0.00**	3.0 ± 0.329	5.35 ± 0.379	0.00**	3.01 ± 0.267	6.12 ± 0.549	0.00**	2.73 ± 0.314	5.61 ± 0.382	0.00**
HOMA-B		146.8 ± 5.902	61.20 ± 4.272	0.00**	145.28 ± 6.558	75.59 ± 5.089	0.00**	142.06 ± 6.0	60.84 ± 2.967	0.00**	154.04 ± 5.898	66.94 ± 5.127	0.00**
Systolic Blood pressure		127.11 ± 1.471	136.08 ± 1.967	0.00**	126.16 ± 1.442	136.61 ± 1.977	0.00^**^	127.44 ± 1.343	136.59± 2.496	0.00**	126.99 ± 1.611	134.45 ± 1.733	0.00**
Diastolic Blood pressure		83.84 ± 1.085	88.43 ± 1.465	0.01*	83.01 ± 1.081	89.32 ± 1.422	0.00**	83.29 ± 0.983	90.68 ± 1.733	0.00**	83.25 ± 1.186	88.44 ± 1.229	0.00**
BMI (kg/m2)		32.7 ± 0.49	35.74 ± 0.853	0.283	32.89 ± 0.548	34.99 ± 0.715	0.02*	33.05 ± 0.509	35.26 ± 0.841	0.02*	33.04 ± 0.535	34.59 ± 0.753	0.098
Total Cholesterol (mg/dL)		189.03 ± 8.925	317.75 ± 10.131	0.00**	187.27 ± 10.004	303.51 ± 8.361	0.00**	196.50 ± 9.212	317.32 ± 9.329	0.00**	188.71 ± 9.844	292.11 ± 10.809	0.00**
LDLc (mg/dL)		130.5 ± 5.896	207.61 ± 7.822	0.00**	125.97± 6.119	205.44± 6.766	0.00**	132.5 ± 5.682	214.3 ± 8.26	0.00**	127.92 ± 6.173	196.09 ± 7.821	0.00**
HDLc (mg/dL)		53.07 ± 1.715	34.55 ± 1.184	0.00**	53.68 ± 1.792	35.95± 1.308	0.00**	52.05 ± 1.672	34.45 ± 1.255	0.00**	55.33 ± 1.71	34.67 ± 1.352	0.00**
TGs (mg/dL)		171.37 ± 7.829	300.98 ± 9.182	0.00**	175.51± 9.018	275.83± 9.425	0.00**	178.92 ± 8.39	300.5± 6.984	0.00**	165.51 ± 8.167	284.08 ± 9.607	0.00**
Alb/Creat Ratio		18.36 ± 0.616	25.47 ± 0.579	0.00**	18.08 ± 0.630	25.02 ± 0.610	0.00**	18.89 ± 0.620	25.14 ± 0.594	0.00**	18.2 ± 0.640	24.28 ± 0.693	0.00**
ALT (IU/L)		45.62 ± 1.495	45.18 ± 1.792	0.849	45.19 ± 1.469	46.02± 1.953	0.738	46.35 ± 1.47	43.07 ± 1.664	0.142	46.52 ± 1.661	43.81 ± 1.472	0.224
AST (IU/L)		43.57 ± 1.483	59.73 ± 6.312	0.016*	47.73 ± 3.293	49.92 ± 2.067	0.644	47.46 ± 2.978	51.43 ± 1.858	0.259	43.23 ± 1.654	56.98 ± 5.077	0.00**
CKMB		23.62 ± 2.44	42.22 ± 4.0	0.00**	20.38 ± 2.01	45.63 ± 4.24	0.00^**^	23.168 ± 2.029	46.45 ± 5.33	0.00**	22.38 ± 2.79	40.44 ± 3.09	0.00**
Troponin		10.40 ± 2.16	34.27 ± 5.03	0.00**	13.40 ± 2.61	25.54± 4.29	0.01*	9.64 ± 1.9	40.2 ± 5.77	0.00**	7.07 ± 1.91	34.78 ± 4.43	0.00**

**Table:** 

Genes			FZD5 mRNA	GTF2I mRNA	has-miR-1976	LINC02210
Spearman’s rho	FZD5 mRNA	Correlation Coefficient	1.000	0.462**	0.632**	-0.651**
		Sig. (2-tailed)	.	<.001	<.001	<.001
		N	167	167	167	167
	GTF2I mRNA	Correlation Coefficient	0.462**	1.000	0.545**	-0.369**
		Sig. (2-tailed)	<.001	.	<.001	<.001
		N	167	167	167	167
	has-miR-1976	Correlation Coefficient	0.632**	0.545**	1.000	-0.456**
		Sig. (2-tailed)	<.001	<.001	.	<.001
		N	167	167	167	167
	LINC02210	Correlation Coefficient	0.651**	-0.369**	-0.456**	1.000
		Sig. (2-tailed)	<.001	<.001	<.001	.
		N	167	167	167	167

** Correlation is significant at the 0.01 level (2-tailed).

In the T2DM+IHD subgroup, pairwise associations within the RNA panel were examined using Spearman’s rank correlation. Positive but non-significant correlations were observed for *FZD5–*hsa-miR-1976 (r = 0.266; *p* < 0.190), *FZD5–*LINC02210 (r = 0.265; *p* < 0.191), *GTF2I–*LINC02210 (r = 0.225; *p* < 0.270), and hsa-miR-1976*–*LINC02210 (r = 0.287; *p* < 0.155). By contrast, *GTF2I* showed inverse correlations with *FZD5* (r = −0.061; *p* < 0.769) and with hsa-miR-1976 (r = −0.137; *p* < 0.505) (**Table 7**). In the T2DM+IHD subgroup, biomarker-clinical correlations appeared attenuated, likely reflecting multifactorial pathophysiology. However, multivariate models and discriminatory performance remained robust, underscoring their complementary value.

**Table 7:** Correlation analysis in T2DM+IHD group.

Genes			FZD5 mRNA	GTF2I mRNA	has-miR-1976	LINC02210
Spearman’s rho	FZD5 mRNA	Correlation Coefficient	1.000	-0.061	0.266	0.265
		Sig. (2-tailed)		0.769	0.190	0.191
		N	26	26	26	26
	GTF2I mRNA	Correlation Coefficient	-0.061	1.000	-0.137	0.225
		Sig. (2-tailed)	0.769	.	0.505	0.270
		N	26	26	26	26
	has-miR-1976	Correlation Coefficient	0.266	-0.137	1.000	0.287
		Sig. (2-tailed)	0.190	0.505	.	0.155
		N	26	26	26	26
	LINC02210	Correlation Coefficient	0.265	0.225	0.287	1.000
		Sig. (2-tailed)	0.191	0.270	0.155	
		N	26	26	26	26

A linear regression analysis was used to evaluate the relationships between RNAs levels across all study groups. *FZD5* mRNA (*p* = 0.001), *GTF2I* mRNA (*p* < 0.001), LINC02210 (*p* = 0.049), CK-MB (*p* < 0.001) and Troponin (*p* < 0.001) were significant predictor, whereas has-miR-1976 (*p* = 0.091) was not significant in the combined analysis (**Table 8**).

**Table 8:** Regression analysis.

Model	Unstandardized coefficients		Standardized coefficients	t	Sig.	95.0% Confidence interval for(B)	
	B	Std. error	Beta			Lower bound	Upper bound
FZD5 mRNA	0.019	0.006	0.238	3.258	0.001	0.007	0.030
GTF2I mRNA	0.031	0.006	0.366	5.244	< 0.001	0.019	0.043
has- miR-1976	0.000	0.000	0.116	1.699	0.091	0.000	0.000
LINC02210	0.000	0.000	-0.130	-1.987	0.049	0.000	0.000
CKMB	0.010	0.003	0.260	3.923	<0.001	0.005	0.015
Troponin	0.010	0.002	0.274	4.133	<0.001	0.005	0.015
(Constant)	1.583	0.101		15.743	<0.001	1.384	1.782

*Linear Regression analysis.

*GTF2I* showed a lower mean Ct in T2DM+IHD (23.5 vs 27.0 in controls), suggesting upregulated expression with intra-assay reproducibility (SD ≤0.27). hsa-miR-1976 showed markedly lower Ct in T2DM+IHD (21.8 vs 29.2 in controls), indicating strong differential expression with slightly higher inter-assay variability (SD = 0.60–0.65), potentially reflecting miRNA stability constraints. Across all targets, technical reproducibility was high with intra-assay CV% <1.4% and inter-assay CV% <1.5% (**Table 9**). has-miR-1976 exhibited the largest fold-change between groups (ΔCt = 7.4), aligning with its proposed role in metabolic regulation (**Supplementary Table S1**).

**Table 9:** Intra-Assay and Inter-Assay Variability for Real-Time PCR.

Gene	Sample group	Intra-assay SD (Ct)^a^	Inter-assay SD (Ct)^b^	Mean Ct	CV%^c^
FZD5	T2DM+IHD	0.26	0.48	24.1	1.08
FZD5	Control	0.29	0.51	26.3	1.10
GTF2I	T2DM+IHD	0.24	0.50	23.5	1.02
GTF2I	Control	0.27	0.55	27.0	1.00
miR-1976	T2DM+IHD	0.30	0.60	21.8	1.38
miR-1976	Control	0.32	0.65	29.2	1.10
LINC02210	T2DM+IHD	0.31	0.58	22.5	1.38
LINC02210	Control	0.27	0.53	25.8	1.05

These findings suggest a potential translational value of the proposed RNA panel in clinical practice. When integrated with existing diagnostic markers such as troponin and HbA1c, this panel could enhance early detection and risk stratification of ischemic heart disease in diabetic patients. The combined use of molecular and conventional biomarkers may improve diagnostic sensitivity and specificity, allowing for better patient monitoring and personalized therapeutic strategies.

## 3 Discussion

Type 2 diabetes mellitus (T2DM) is now regarded not only as a metabolic disorder but also as an independent driver of cardiovascular risk, most notably ischemic heart disease (IHD), in which inadequate myocardial perfusion culminates in tissue injury. Furthermore, mounting evidence indicates that the underlying mechanisms of both T2DM and ischemic heart disease are closely linked through inflammatory processes and oxidative stress, which are exacerbated by mitochondrial dysfunction and cellular apoptotic pathways, as highlighted in the literature on cell-fate regulation (21).

Whether regional adiposity is linked to cardiovascular disease (CVD) risk and mortality in individuals with type 2 diabetes (T2DM) remains largely unclear, despite their characteristic shifts in fat distribution and elevated CVD risk (22).

These links likely reflect a multifactorial interaction between genetic variation and epigenetic regulation that shapes RNA-mediated regulation of gene expression. Growing evidence indicates that disturbances within RNA regulatory networks are central to the pathogenesis of T2DM and its complications. By clarifying how genetic variations and epigenetic modifications affect gene expression, we can better elucidate the molecular mechanisms that drive T2DM and its downstream cardiovascular risks. Our objective was to determine the discriminatory performance of a molecular RNA panel comprising *FZD5* and *GTF2I* for the early identification of ischemic heart disease in individuals with type 2 diabetes mellitus.

Multiple risk loci linked to insulin resistance and lipid metabolism have been reported, and these variants not only increase susceptibility to type 2 diabetes but also heighten vulnerability to cardiovascular outcomes. For example, variants that impair endothelial-cell function can lead to impaired vascular responses, as evidenced by the common pathology of diabetic panvascular disease (DPD), in which macrovascular and microvascular complications often emerge concurrently in individuals with diabetes, suggesting a shared or overlapping pathogenic timeline that may accelerate systemic deterioration (23). In addition, underlying genetic predisposition can amplify endoplasmic-reticulum (ER) stress signaling implicated in T2DM pathobiology, thereby aggravating cellular dysfunction and promoting progression toward ischemic cardiovascular events (24).

We first constructed a regulatory network spanning mRNA/miRNA/lncRNA interactions relevant to crosstalk in T2DM with IHD using computational analyses. We then quantified serum levels of network components in cases and controls to appraise their capacity for early risk stratification and discriminatory assessment (CVD). A substantial subset of the mapped genes was associated with IHD and T2DM. Prior work has shown increased methylation at the *FZD5* promoter in T2DM patients and IHD, consistent with reports implicating *FZD5* in diabetic vasculopathy (25). Concordantly, our data revealed elevated *FZD5* mRNA in patients with T2DM+IHD.

Independent reports indicate that increased methylation of *GTF2I* is associated with a higher subsequent risk of myocardial infarction and coronary heart disease (26). This aligns with our findings, which showed an elevated *GTF2I* mRNA in the T2DM+IHD group, suggesting its involvement in the development of IHD among patient with T2DM patients.MicroRNAs have emerged as informative biomarkers for diabetes and its sequelae. Their reliable detection in circulating biofluids has driven extensive investigation into disease-specific expression profiles and molecular stability. In particular, miR-92a, miR-503, and miR-126 modulate angiogenic pathways, processes that are essential for myocardial repair after ischemic injury (11).

These observations accord with our findings, which showed upregulation of *hsa-miR-1976* and support its role as a putative epigenetic activator of the *FZD5/GTF2I* axis. This interpretation is consistent with recent reports that certain miRNAs can engage promoter regions and enhance transcription via RNA-activation (RNAs). To our knowledge, this is the first description linking *hsa-miR-1976* to type 2 diabetes complicated by ischemic heart disease.

Multiple reports highlight the central regulatory functions of lncRNAs across the initiation and progression of T2DM with coexisting IHD (20). Crosstalk among these transcripts appears to coordinate gene programs relevant to IHD pathogenesis and positions lncRNAs as candidate biomarkers for early detection and risk prediction in patients with T2DM. Consistently, specific lncRNAs exhibit discriminatory translational potential in diabetes complications, serving as molecular readouts of disease onset, trajectory, and tissue specificity. Supporting this concept, Geng et al.” (2024) reported reduced levels of *TINCR* and *HOTAIR* in serum and myocardial tissue from individuals with diabetic complications, which discriminated cases from healthy controls.

Notably, our data indicate that LINC02210 functions as a putative network-associated regulator within the *FZD5/GTF2I/*hsa-miR-1976 network. To our knowledge, LINC02210 has not been previously linked to type 2 diabetes or ischemic heart disease. In this cohort, circulating LINC02210 levels were lower in T2DM+IHD than in either controls or T2DM alone, and yielded discriminatory decision thresholds capable of separating T2DM+IHD vs controls, T2DM vs IHD and T2DM+IHD vs T2DM.

LINC02210’s inverse correlations with angiogenesis-related genes (*FZD5, GTF2I*) and discriminatory performance in advanced disease stages (AUC > 0.97) suggest it may modulate vascular remodeling. Ongoing work is testing its direct role in endothelial dysfunction and plaque stability. While LINC02210 demonstrates disease-specific expression patterns, its functional role requires validation in ongoing studies.

The evaluated angiogenesis-linked RNA signature showed group-dependent expression. Levels of *FZD5* and *GTF2I* mRNAs, together with hsa-miR-1976, rose stepwise from controls to T2DM and IHD, with peak abundances observed in the T2DM+IHD cohort. Conversely, LINC02210 displayed a graded decline across the same sequence, reaching its lowest concentration in T2DM+IHD. Taken together, these trajectories support the feasibility of this circulating coding/non-coding RNA panel as an early-detection aid for ischemic heart disease in the context of type 2 diabetes. The weaker correlations in T2DM+IHD highlight the need for nonlinear or pathway-specific analyses in advanced disease, which will be pursued in future work.

Relative to the T2DM+IHD cohort, the T2DM group showed higher hsa-miR-1976 and lower LINC02210 expression. Alongside CK-MB and troponin, these noncoding RNA readouts could assist in distinguishing IHD status among patients with T2DM. This interpretation aligns with Ortiz-Martín et al. (2022), who proposed that serum biomarkers can complement or in some settings substitute for traditional analytes for diabetes detection and follow-up. In our data, *FZD5, GTF2I*, hsa-miR-1976, and LINC02210 effectively differentiated T2DM from T2DM+IHD, consistent with prior reports identifying ncRNA signatures as candidate predictors of IHD in diabetes (25–27). While our models show strong discriminatory performance, external validation is required to confirm generalizability; We are actively collaborating with independent cohorts to address this limitation. Previously, our group likewise reported discriminatory utility for a panel comprising *MEMM173* and *CHUK* mRNAs together with hsa-miR-611, -5192, and -1976 in diabetes and cardiovascular disease (6).

The RNA panel (AUC = 0.94) outperformed Troponin-I (AUC = 0.78) and HbA1c (AUC = 0.85) in discriminating T2DM-IHD from controls). Integrating RNA biomarkers with troponin/HbA1c may improve early risk stratification for ischemic events in diabetic populations.

Limitations. This study has several limitations that should be considered when interpreting the findings. To minimize bias, we focused on angiogenesis-related genes with established roles in T2DM/IHD pathways and validated qPCR results in triplicate, achieving low technical, variability (CV < 5%). Nevertheless, the pilot nature of the work and the modest sample sizes in the IHD and T2DM+IHD groups may limit precision and generalizability. Although major confounders were adjusted for, residual confounding from unmeasured factors (e.g., dietary habits*&* drug therapy) may persist; sensitivity analyses supported the robustness of the main signals but cannot fully exclude such effects. *We plan to expand this pilot to a larger cohort with orthogonal validation via wider transcript profiling &protein-level assays*.

The age cutoff of 35 years was selected to minimize age-related comorbidities and to focus on early molecular changes in T2DM and IHD, in line with regional epidemiology; this strengthens internal validity but constrains extrapolation to older populations. Despite statistical matching on age and sex, the absolute sex ratios reflect real-world clinical demographics and could introduce subtle confounding, motivating sex-stratified designs and covariate-adjusted models in future work.

Because multiple genes were evaluated, a risk of type I error remains despite adjusted analyses; larger, prespecified cohorts with formal multiple-testing control are warranted. The putative regulatory role of LINC*02210*, inferred from network centrality and correlations with angiogenic markers, requires confirmation in targeted functional experiments. Finally, although the RNA panel shows encouraging case–control discrimination, clinical validity should be assessed in prospective, blinded, longitudinal cohorts with orthogonal transcriptomic and protein-level assays.

In conclusion, we identify a candidate angiogenesis related RNA panel *FZD5*, *GTF2I* mRNAs, *hsa-miR-1976*, and the lncRNA LINC02210 that is associated with T2DM complicated by IHD and shows concordance with serum clinical measures reflecting the transition from T2DM to T2DM+IHD. These associations are correlative and do not establish causality; prospective validation in larger, age-diverse cohorts, alongside functional studies to delineate gene-specific contributions to IHD risk in T2DM, is required.

## 4 Materials and methods

### 4.1 Bioinformatics-based construction of the RNA regulatory network

We performed an in silico screen to identify differentially expressed coding and noncoding RNAs relevant to type 2 diabetes mellitus (T2DM) and ischemic heart disease (IHD). Microarray expression datasets were obtained from the NCBI Gene Expression Omnibus (GEO; https://www.ncbi.nlm.nih.gov/geo/).

#### 4.1.1 Acquisition of available datasets

High-throughput microarray datasets for diabetic nephropathy and acute coronary syndrome (ACS) were retrieved from NCBI GEO (https://www.ncbi.nlm.nih.gov/geo/, accessed July 2021) (28). Searches were limited to Homo sapiens and experimental studies comparing patients with diabetic nephropathy or ACS against healthy controls. As a result, two datasets were obtained:GSE30122 (29) and GSE19339 (30), were obtained. The GSE30122 dataset contains 19 diabetic kidney samples and 50 healthy control kidney samples, based on the *GPL571* Affymetrix Human Genome U133A 2.0 Array platform. GSE19339 comprises 4 thrombus leukocyte samples from ACS cases and 4 peripheral blood leukocyte samples from healthy controls, generated on the GPL570 Affymetrix Human Genome U133 Plus 2.0 Array platform.

#### 4.1.2 Differential expression analysis

Microarray profiles from GSE30122 and GSE19339 were analyzed using the GEO2R web portal (https://www.ncbi.nlm.nih.gov/geo/geo2r/; accessed July 2021) to identify differentially expressed genes (DEGs) among the groups. GEO2R is an online interface built on the R language limma package (31). Significance thresholds were FDR < 0.05 together with p < 0.05. Probes lacking an assigned gene symbol were excluded prior to downstream analyses of the resulting DEGs. DEGs were identified using |logFC| > 0.5 and p < 0.05, followed by FDR correction (q < 0.1). Functional enrichment required q < 0.05. Thresholds were selected to harmonize statistical rigor with biological plausibility.

#### 4.1.3 Identification of common DEGs

DEGs from both datasets (GSE30122 and GSE19339) were intersected using an online Venn diagram (http://bioinformatics.psb.ugent.be/webtools/Venn/) to obtain the shared gene set. This overlap was considered the set of DEGs implicated in both diabetic nephropathy and acute coronary syndrome progression.

#### 4.1.4 Enrichment analyses of common DEGs

To determine which biological processes (BP) and pathways were overrepresented among the shared DEGs, we performed GO–BP and pathway enrichment using FunRich (http://www.funrich.org/; v3.1.3, accessed Jul 2021) (32). A p-value of <0.05 was considered indicative of enrichment. The biological classification of the common DEGs was subsequently filtered, focusing on the highly significant BP terms associated with angiogenesis.

#### 4.1.5 Protein–protein interaction network analysis

To map potential interactions among proteins encoded by the filtered DEGs and to identify hub nodes, angiogenesis-related DEGs from the enrichment step were queried in STRING (https://string-db.org/; v12, accessed July 2023) (33). Only edges with combined score > 0.15 were retained for network construction. The resulting PPI networks was then visualized using Cytoscape software (version 3.10.2). Topological metrics were then computed with the CentiScaPe app (34) and the degree (number of connections) of each node was calculated; genes with degree >5 were defined as hub genes.

#### 4.1.6 Selection of candidate genes

Biomarkers (mRNAs and miRNAs) were selected through a structured, multi-step integrated bioinformatics pipeline and previous literature validation studies designed to prioritizing relevance to diabetic nephropathy or acute coronary pathogenesis, functional annotations, and prior evidence of differential expression (**Supplementary Table S1**).

From the hub set, we prioritized *FZD5* (Frizzled class receptor 5) and *GTF2I* (General Transcription Factor II-I) to assemble a targeted co-regulatory network. Support for their relevance derives from prior studies (25–27) and from public resources the Comparative Toxicogenomics Database (http://ctdbase.org/) and Gene Cards (https://www.genecards.org/; accessed October 2024) which annotate these genes as linked to angiogenesis and implicated in acute coronary syndrome and diabetic nephropathy progression. The curated genes were subsequently submitted to STRING to construct the protein–protein interaction (PPI) network.

#### 4.1.7 Prediction of candidate microRNAs

Predicted interactions between miRNAs and the selected candidate genes were generated using miRWalk 3.0 (http://mirwalk.umm.uni-heidelberg.de/). Functional implications of the selected miRNA were then evaluated with DIANA tools miRPath v4 module (http://www.microrna.gr/miRPathv4), which tests enrichment of its targets across defined biological pathways.

#### 4.1.8 Prediction of candidate long noncoding RNAs

LncBase predicted version 3 (DIANA Tools - miRNA-lncRNA interactions (uth.gr) was used to predict interactions between long noncoding RNAs (lncRNAs) and the chosen candidate genes, Additional annotation and verification were obtained from Gene card(GeneCards - Human Genes | Gene Database | Gene Search). We confirm the selected lncRNA from another database (LNCipedia database) (https://ngdc.cncb.ac.cn/databasecommons/database/id/24).

### 4.2 Participants and study groups

The study enrolled 167 participants distributed into four groups: 56 Patients who fulfilled the American Diabetes Association’s (ADA) T2DM criterion and had no cardiovascular disease, 25 Patients who had a cardiovascular disease only, 26 Patients who fulfilled the American Diabetes Association’s (ADA) T2DM criterion and has cardiovascular disease and 60 Individuals with normal blood glucose levels who have never had diabetes or any kind and Cardiovascular diseases.

The study cases were enrolled from Cardiology and Endocrinology Department Ain Shams University. The study protocol was approved by the Research Ethics Committee, Faculty of Medicine, Ain Shams University (FMASU R 42/2024). Written informed consent was obtained from all participants in accordance with the Declaration of Helsinki after clear explanation of the study aims, procedures, and potential risks. Data confidentiality was maintained throughout to safeguard participant privacy.

Exclusion criteria of the study included patients with other kinds of diabetes mellitus, severe liver dysfunction, acute infections, active neoplasm. pregnancy patients, Breast feeding patients with mental disorder, autoimmune disease, Patients that are uncooperative and refuse to give consent, Patients that are less than 35 years old and Patients that are related to angiogenesis disease such as numerous malignant, inflammatory, infectious and immune disorders.

Venous blood was obtained from all participants. Serum was separated by centrifugation at 4, 000 rpm for 20 min, aliquoted, and stored at −80 °C until analysis. A multifunctional biochemistry analyzer (AU680, Beckman Coulter Inc., Kraemer Blvd., Brea, CA 92821, USA) was used to assess serum lipid profile, liver function tests, CKMB, Troponin, HBA1C, Insulin level, post prandial glucose and fasting glucose. HOMA-IR calculated as (Fasting insulin (μU/L) × fasting glucose (nmol/L)/22.5) (35).

### 4.3 RNA isolation and cDNA preparation

Total RNA was isolated from serum using the miRNeasy Mini Kit (Qiagen, Hilden, Germany, cat. no. 217084) according to the manufacturer’s instructions. RNA yield and purity were quantified on a Qubit 3.0 Fluorometer (Invitrogen, Life Technologies, Malaysia) with the Qubit™ dsDNA HS Assay Kit and the Qubit™ RNA HS Assay Kit (cat. nos. Q32851 and Q32852, respectively). cDNA was then synthesized from the purified RNA using a Rotor gene Thermal cycler (Thermo Electron Waltham, MA) and the QuantiTect Reverse Transcription Kit for mRNA and lncRNA (Qiagen, Hilden, Germany, cat. no. 205311) and the miRCURY LNA RT Kit (Qiagen, Hilden, Germany, cat. no. 339340) for miRNA in reference to the kit’s protocol.

### 4.4 Quantitative RT-PCR analysis of target mRNAs/miRNA/lncRNA

Prior studies demonstrating *ACTB, GAPDH* showed the most stable expression. stable expression in human blood and vascular tissues under metabolic stress (PMID: 38766348, PMID: 37223013). We employed geometric mean normalization (*GAPDH* + *ACTB*) to minimize individual gene fluctuations, as recommended for metabolic disease studies (36). Reference gene stability and assay performance are summarized in **Supplementary Table S2**.

mRNA targets (*FZD5*, *GTF2I*) were quantified using gene-specific primer assays in combination with the QuantiTect Multiplex PCR Kit (Qiagen, Hilden, Germany, cat. no. 249900; assay IDs QT00200886 and QT01677305), with *GAPDH* and *ACTB* serving as internal references. For miRNA measurements, hsa-miR-1976 was amplified with the miRCURY LNA SYBR Green PCR Kit (Qiagen, cat. no. 339345) and the corresponding assay (Cat. No. 339350; ID: ZP00000388), and expression was normalized to *SNORD44*. LINC02210 (lncRNA) levels were determined using the RT2 lncRNA qPCR Assay (Qiagen, cat. no. 330701), with *GAPDH* as the reference control. Thermal cycling conditions were 95 °C for 2 min, followed by 45 cycles of 95 °C for 5 s and 60 °C for 10 s. Relative expression was computed by the Livak method (RQ = 2^−ΔΔCt) (37), and reactions were run on an Applied Biosystems 7500 Fast System (37). All primer assays utilized in this study were sourced from Qiagen, Germany (**Supplementary Table S3**).

### 4.5 Statistical analysis

All analyses were conducted in SPSS v29 (IBM, Chicago, USA). Continuous variables are summarized as median [IQR] for non-normally distributed data and mean ± SD for normally distributed data. Normality was examined with the Shapiro–Wilk test. Between-group comparisons used Kruskal–Wallis with Dunn’s *post-hoc* procedure for nonparametric outcomes, and one-way ANOVA with Tukey’s *post-hoc* test for parametric outcomes. Demographic characteristics and clinical predictors of T2DM+IHD were evaluated within this framework. Two-sided p < 0.05 was considered statistically significant. Multicollinearity was assessed via correlation matrices. Covariates were selected *a priori* based on clinical relevance.

### 4.6 Measures to overcome risks of overfitting

#### 4.6.1 Feature selection rationale

Gene candidates were prioritized through a biology-driven strategy focusing on hypoxia-responsive angiogenesis pathways implicated in T2DM and ischemic heart disease (IHD) pathogenesis. Targets such as *FZD5* were selected based on pathway enrichment analyses and prior literature evidence of their roles in endothelial dysfunction (38). Biomarker inclusion criteria required both statistical significance (adjusted p<0.05) and biological relevance (≥2-fold differential expression), ensuring alignment with disease mechanisms while minimizing false discovery.

#### 4.6.2 Experimental design

Technical reproducibility was ensured through triplicate PCR measurements for all samples, achieving coefficient of variation (CV) values <1.5% for cycle threshold (Ct) values (**Table 9**). Biological replicates were incorporated to account for inter-individual variability inherent in human studies. Statistical analyses employed ANOVA & Kruskal-Wallis tests (for non-normally distributed data) with *post-hoc* correction to address multiple comparisons. *A priori* power analysis (α=0.05, β=0.20) confirmed adequate sample size to detect ≥2-fold expression differences, aligning with clinically relevant thresholds in metabolic disease research.

#### 4.6.3 Reproducibility metrics

Stringent quality control included evaluation of intra-assay (within-run) and inter-assay (across-run) variability, with Ct standard deviations maintained at ≤0.33 and ≤0.65, respectively (**Table 9**). These metrics, combined with primer efficiencies of 90–105% (**Supplementary Table S3**), met MIQE guidelines for qPCR reliability. The low CV% values (<1.5%) across all targets underscore the technical precision of our experimental workflow.

## Data Availability

The original contributions presented in the study are included in the article/**Supplementary Material**. Further inquiries can be directed to the corresponding author.
