# Supplementary material for: Genetic/epigenetic RNA dysregulation in type 2 diabetes mellitus complicated with ischemic heart disease
Source: Front Endocrinol (Lausanne). 2025 Oct 31;16:1687145. doi: 10.3389/fendo.2025.1687145 (PMC12615181; doi:10.3389/fendo.2025.1687145)
Supplement: Supplementary file 1 [file DataSheet1.docx]

**
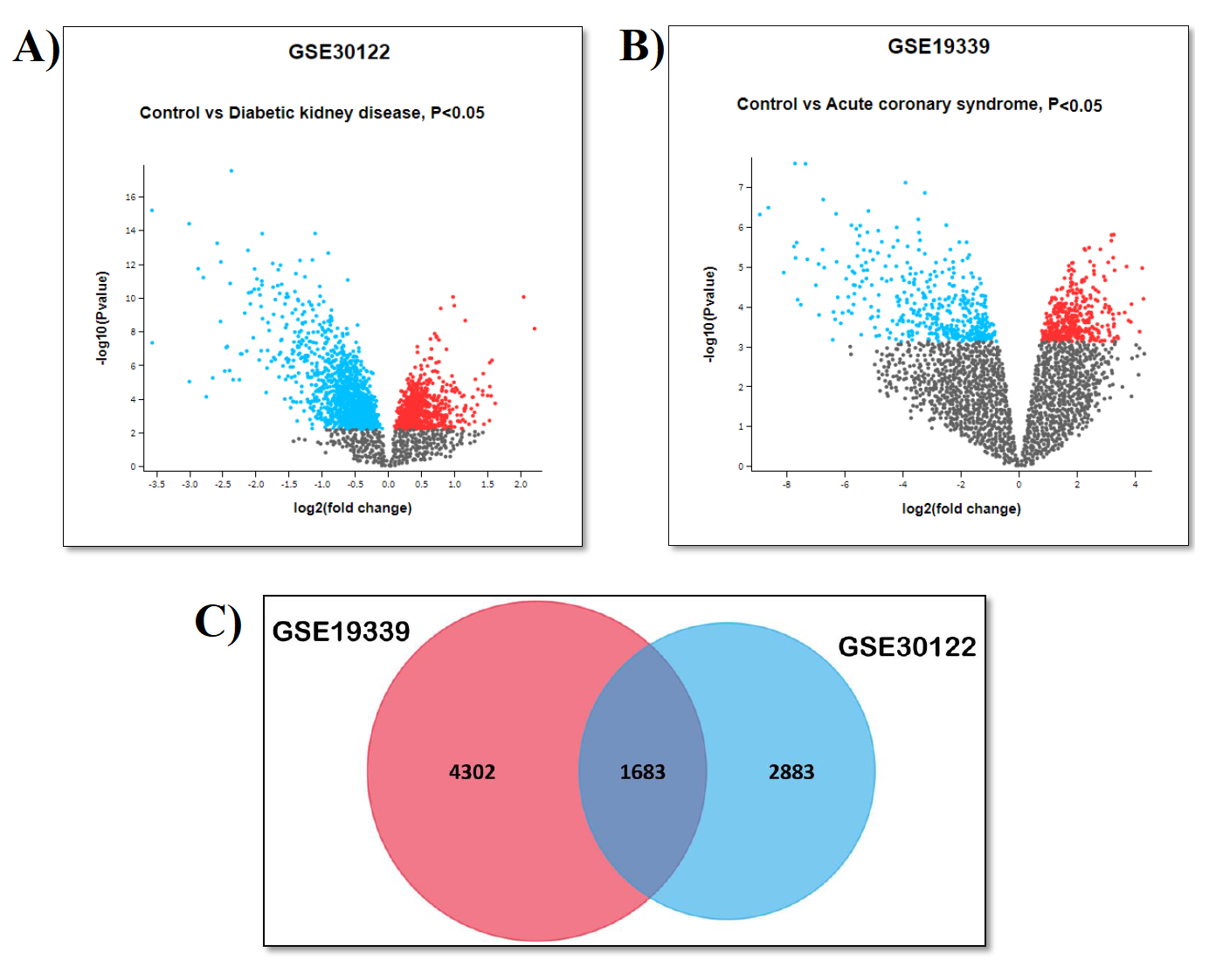
Figures**

**Supplementary Figure 1**: Differential expression analysis. **(A & B)**: Volcano plots for identification of differentially expressed genes (DEGs) in the GSE30122 and GSE19339 datasets, respectively. Each point represents a gene from the individual dataset. Data points highlighted in red represent upregulated genes and blue points indicate downregulated genes. The black dots represent genes without significant difference in expression. **(C)**: Venn diagram for DEGs between the two GEO datasets.


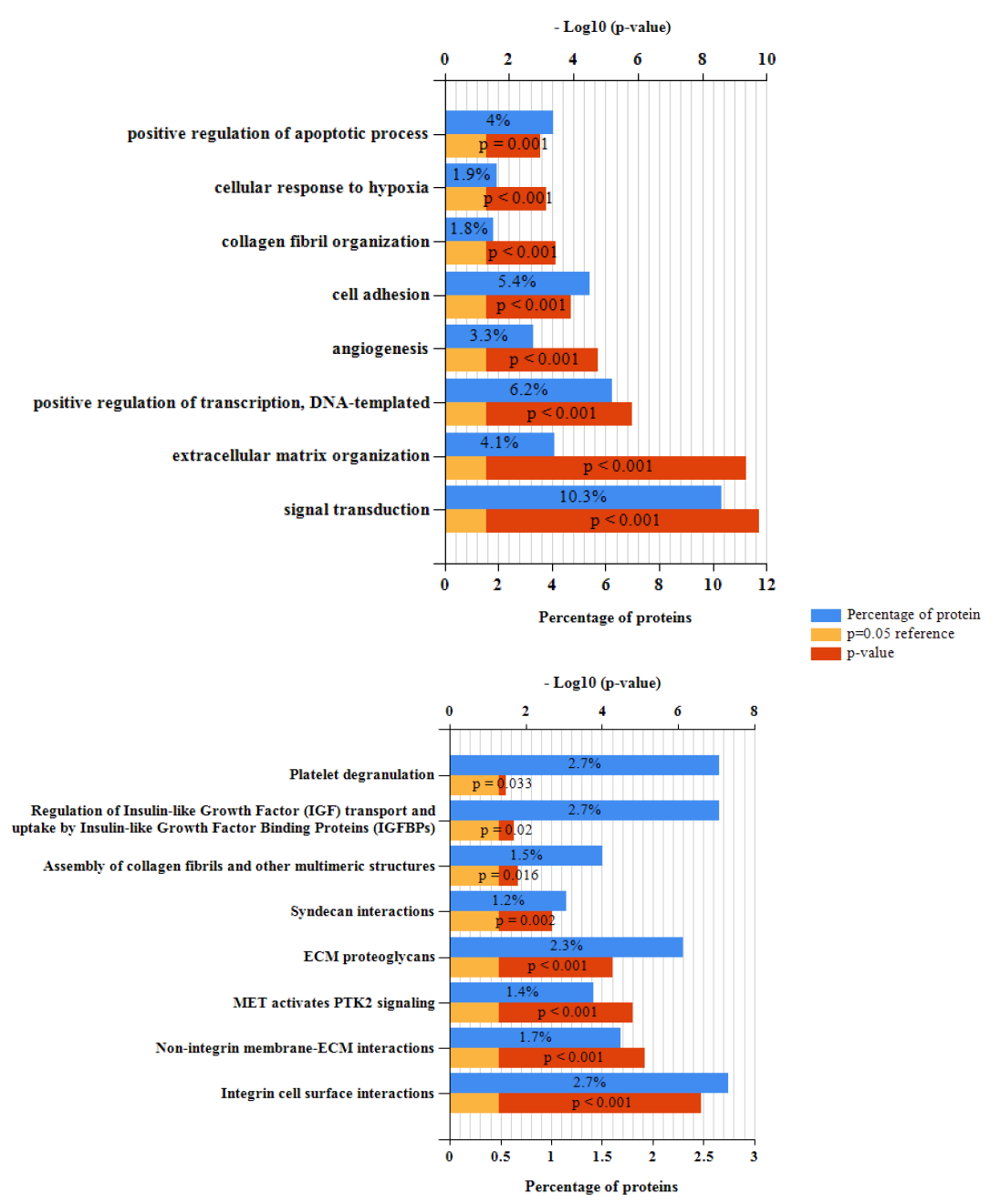
 **Supplementary Figure 2:** Top 8 items of **A)** Biological processes and **B)** Reactome pathways for the common DEGs shown in the bar chart according to adjust *p* value.

**
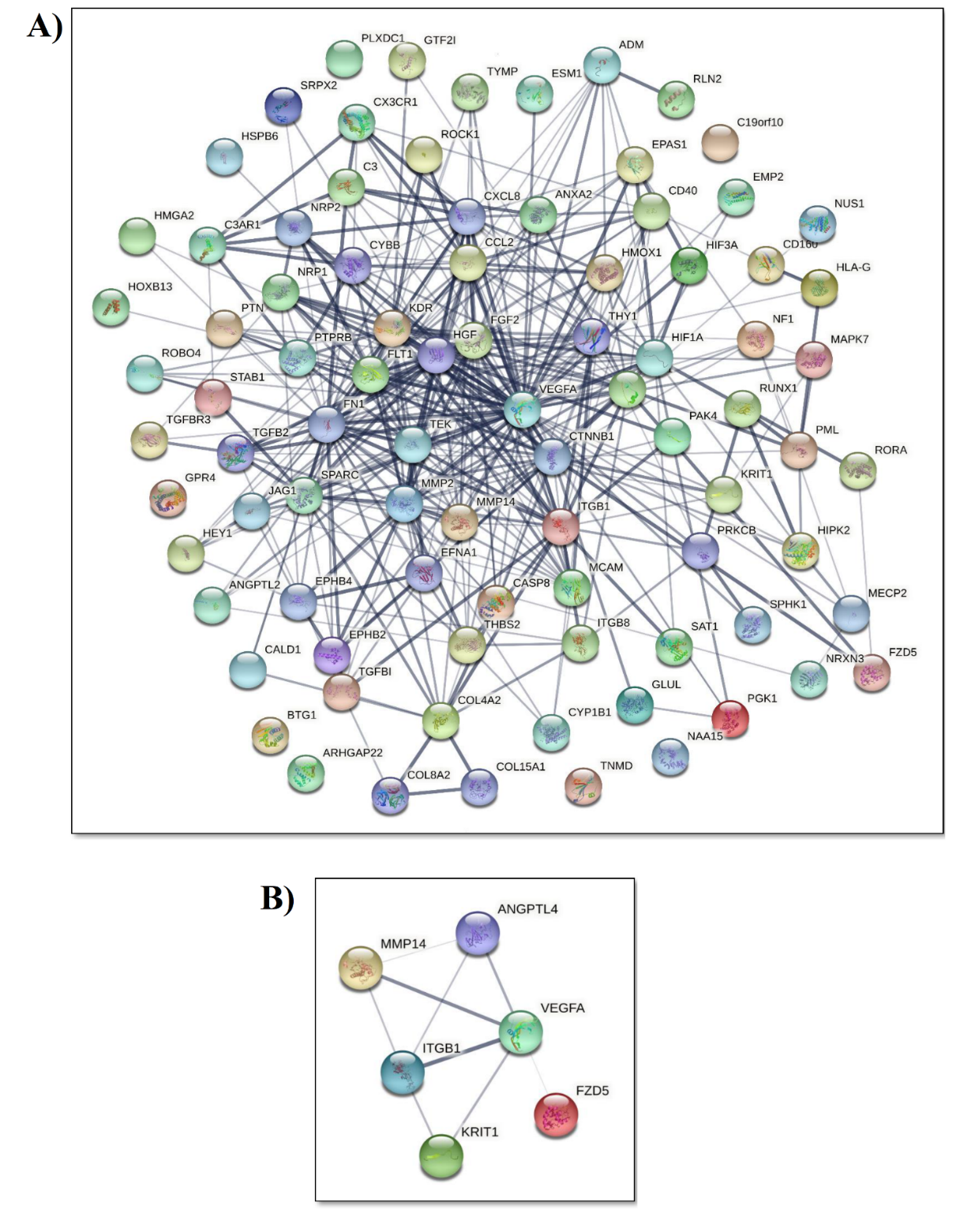
** **Supplementary Figure 3** :The protein–protein interaction networks that were obtained by using String tool. (A) The filtered angiogenesis-related gene. (B) The selected candidate hub genes. (<https://string-db.org/> ; version 12.0, accessed on Oct 2024).

**Supplementary Figure 4**: Validation for the candidate FZD5 gene relation with IHD, T2DM and angiogenesis by Comparative Toxicogenomics Database (<http://ctdbase.org/>, accessed on Oct 2024).

**
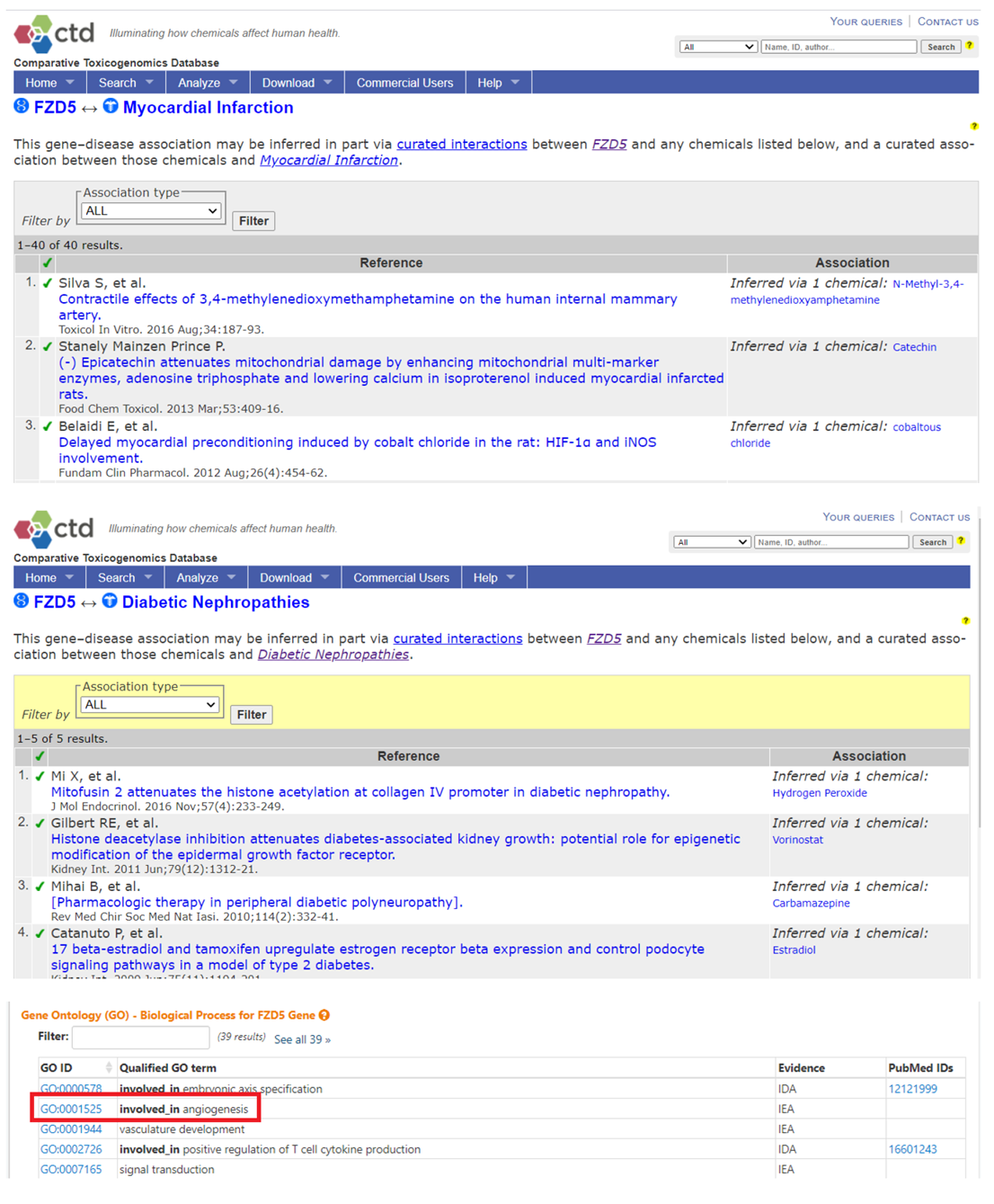
**

**Supplementary Figure 5**: Validation for the candidate GTF2I gene relation with IHD, T2DM and angiogenesis by Comparative Toxicogenomics Database (<http://ctdbase.org/>, accessed on Oct 2024).


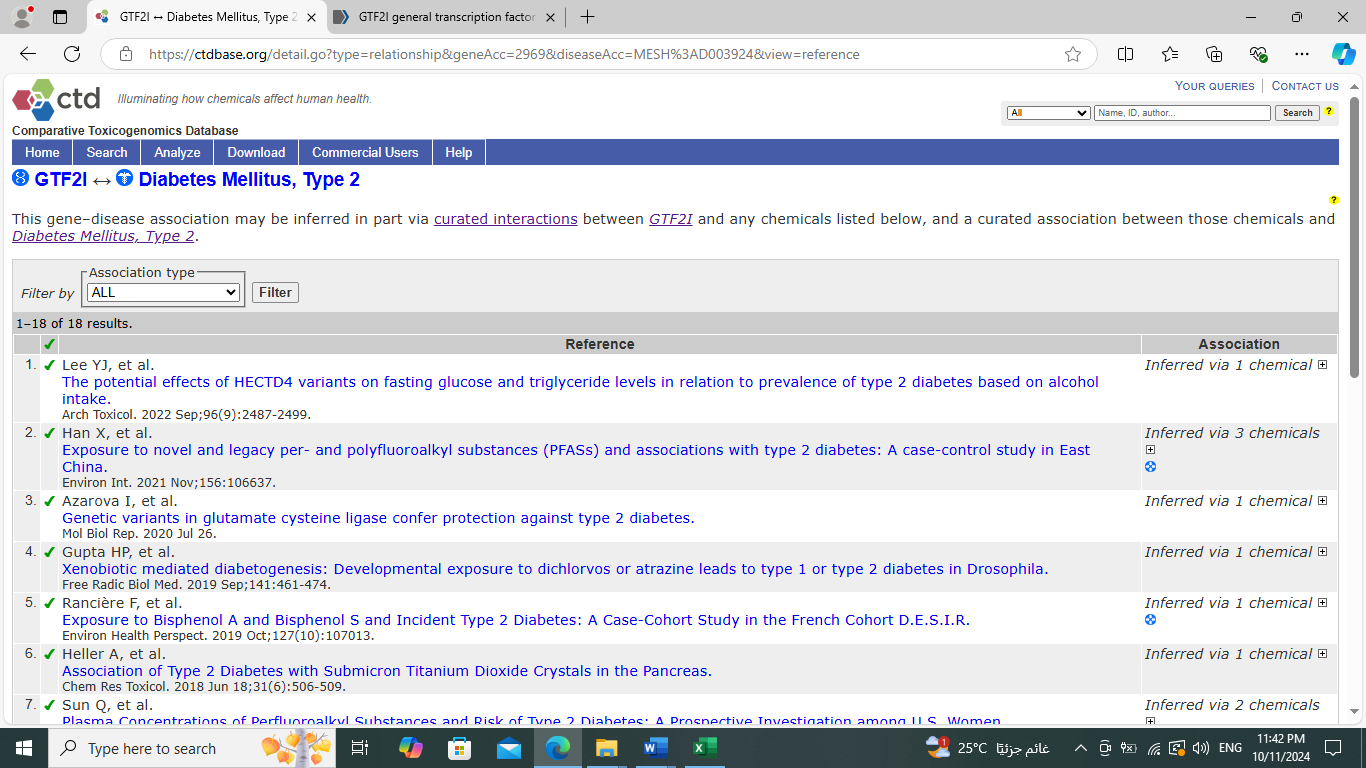

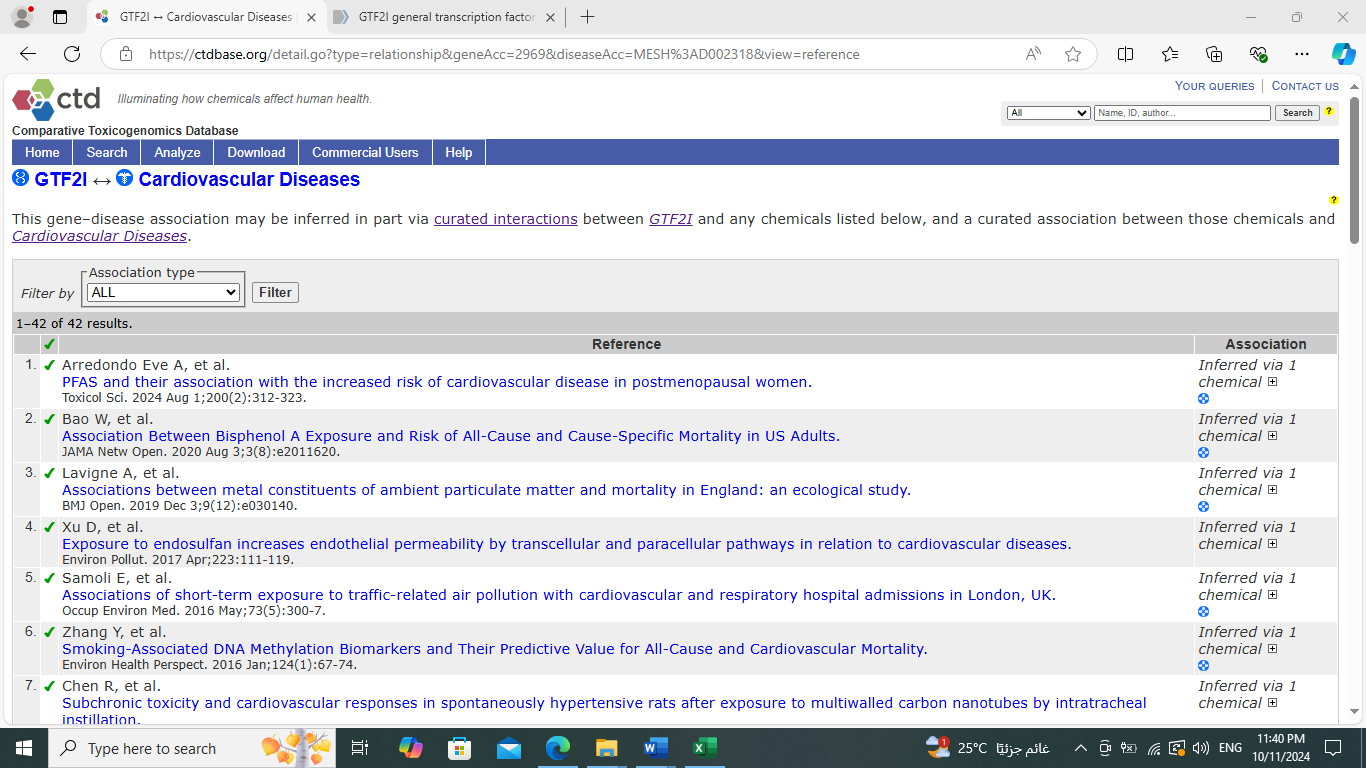

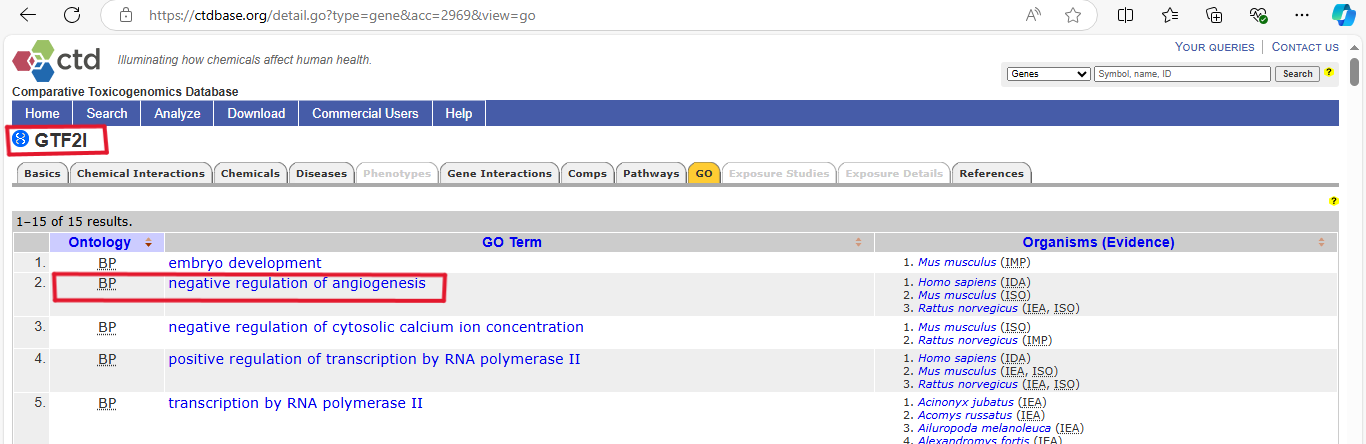


**Supplementary Figure 6**: Screenshot for the results retrieved after querying the interaction of the selected genes with has-miR-1976 with score ˃ 0.85 by use (<http://mirwalk.umm.uni-heidelberg.de/>, version.3.0, accessed on Oct 2024).


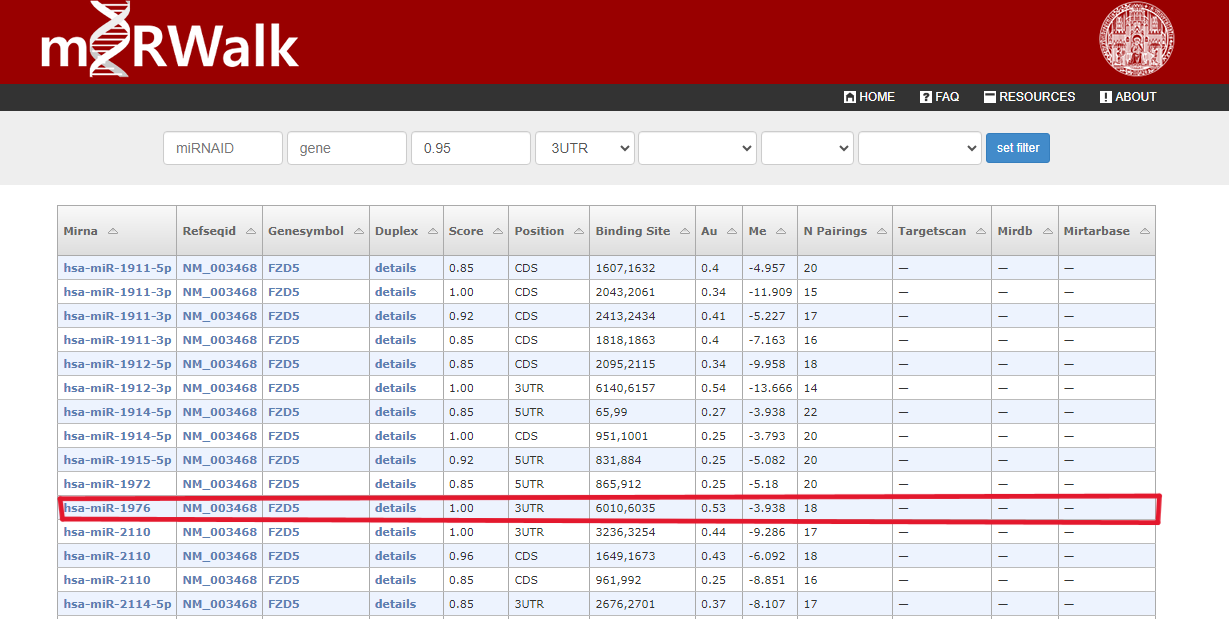

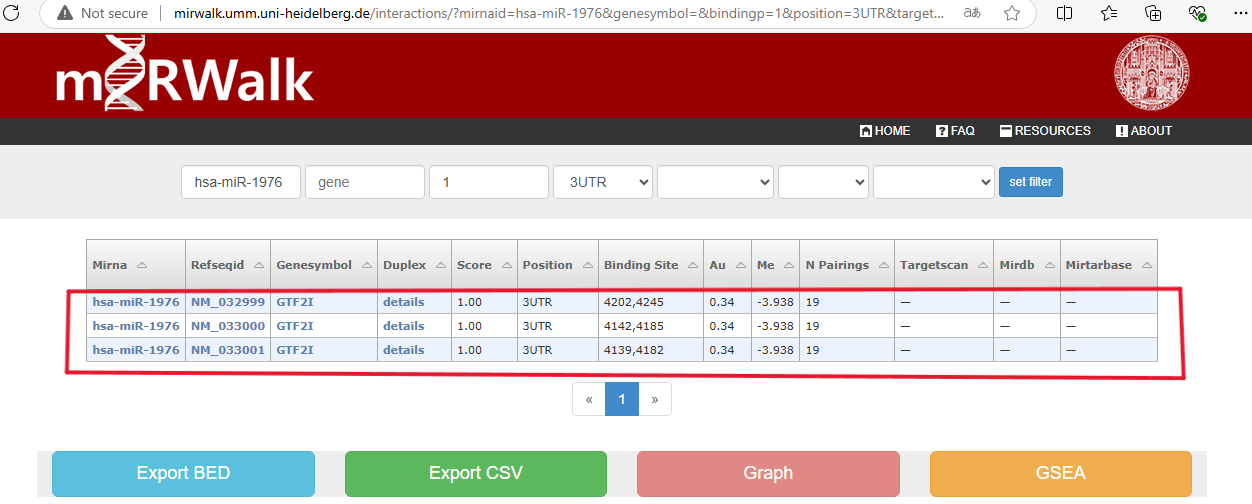


**Supplementary Figure 7**: Screenshot show that has-miR-1976 was strongly linked to acute coronary syndrome and diabetic nephropathy progression and confirmed by ([miRPath v4.0 - Default](http://62.217.122.229:3838/app/miRPathv4) , accessed on Oct 2024)


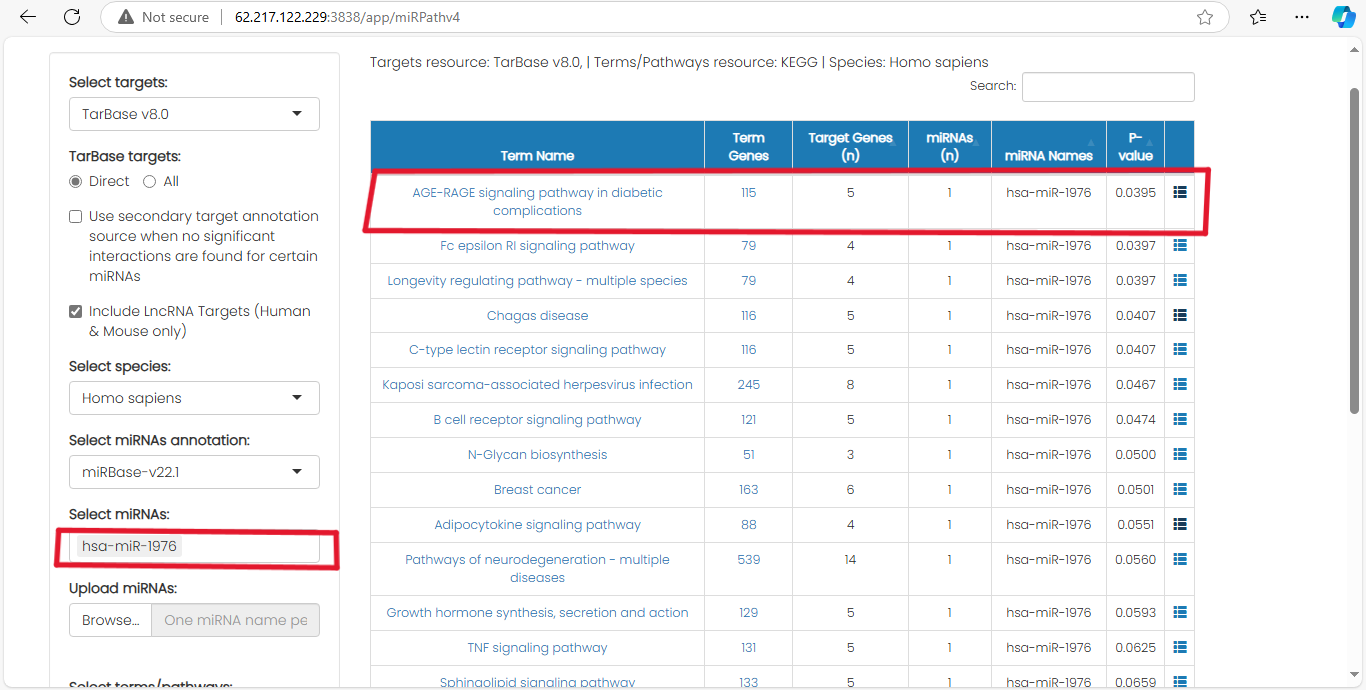

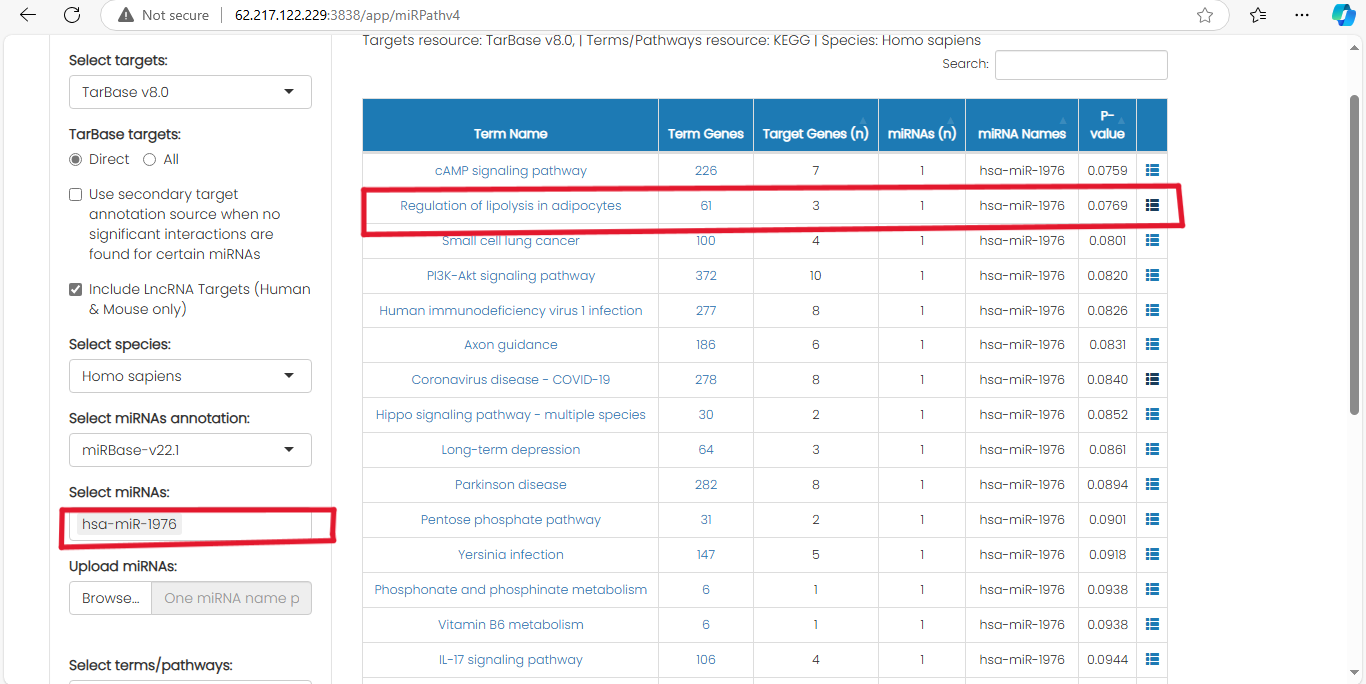

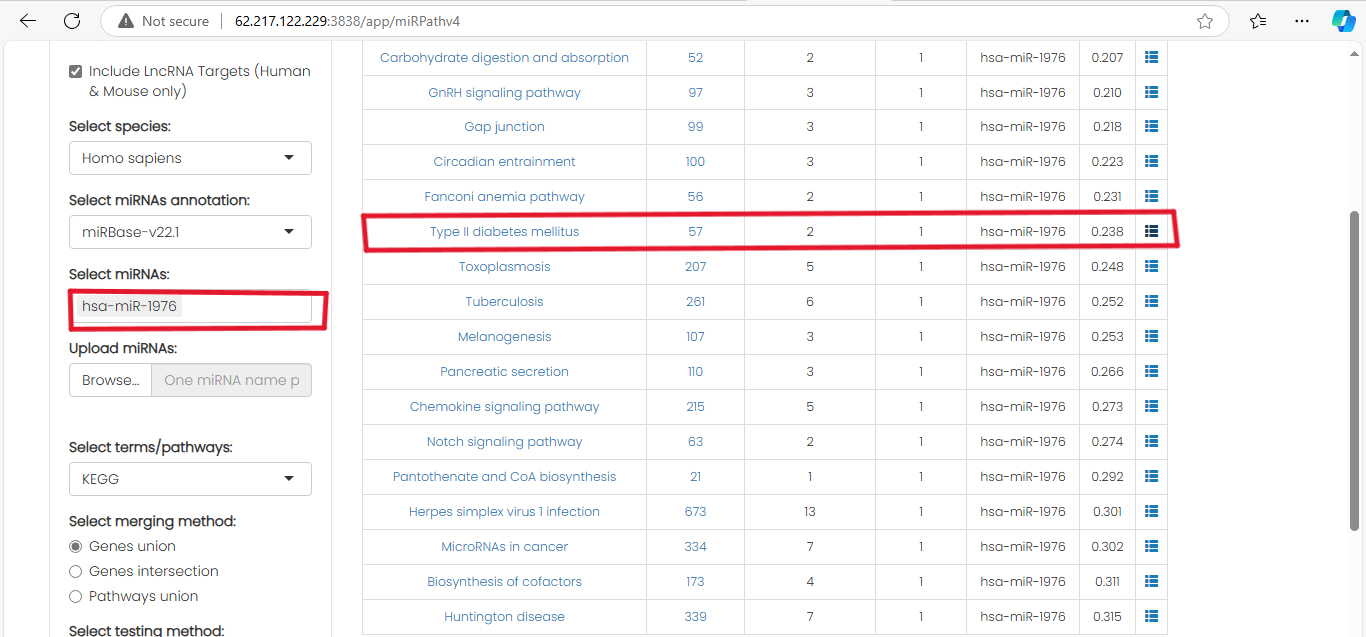


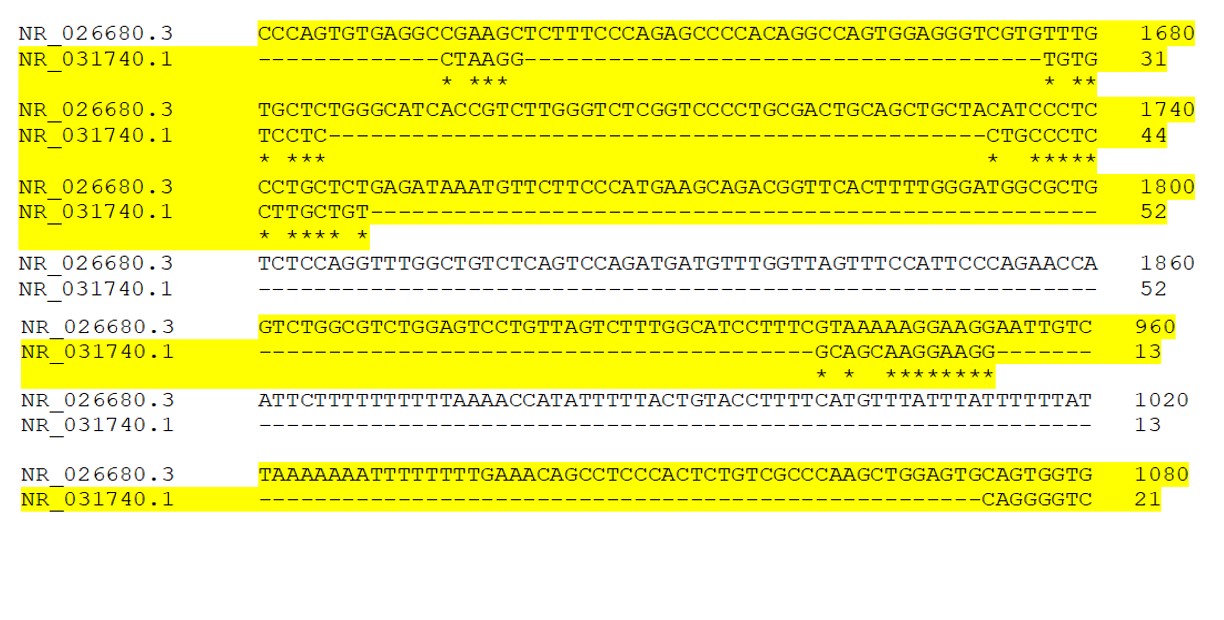


**LINC02210**

**hsa-miR-1976**

**Supplementary Figure 8**: Screenshot show that Claustral multiple sequence alignment to verify interaction between has-miR-1976 miRNA and LINC02210 long non coding RNA (<https://www.ebi.ac.uk/jdispatcher/msa/clustalo>), accessed on Oct 2024)
