# Supplementary material for: Genetic/epigenetic RNA dysregulation in type 2 diabetes mellitus complicated with ischemic heart disease
Source: Front Endocrinol (Lausanne). 2025 Oct 31;16:1687145. doi: 10.3389/fendo.2025.1687145 (PMC12615181; doi:10.3389/fendo.2025.1687145)
Supplement: Supplementary file 2 [file DataSheet2.docx]

**Supplementary Table 1:** The criteria that were used to select only those specific genes and miRNA.

| **Criteria** | **Selection Process/Rationale** | **Key Parameters/Examples** |
| --- | --- | --- |
| **Differential Expression Analysis**  **(to ensure that the chosen genes closely linked to diabetic nephropathy or acute coronary syndrome** | - **GEO Datasets**: Filtered using keywords ("T2DM," " diabetic nephropathy or acute coronary syndrome") and inclusion criteria (array data, ≥4 samples per group). - **DEG Identification**: Applied GEO2R/limma (R package) with thresholds: \|logFC\| > 0.5, *p* < 0.05. | • Datasets: Publicly available raw data (Supplementary Table S1,S2). • Examples: DEGs like *FZD5 , GTF2I*. |
| **Functional Relevance**  **to verify that the chosen genes linked to Angiogenesis & hypoxia as crucial molecular pathways implicated in** diabetic nephropathy or acute coronary syndrome **pathogenesis** | - **Gene Ontology (GO)**: Filtered via FunRich  . A p-value of <0.05 was considered  to indicate a significantly enriched angiogenesis or hypoxia or cell death pathway. - **PPI Networks**: STRING database identified hub genes (medium confidence: 0.4; FDR: 5%). | • GO categories: Insulin signaling , inflammation ,angiogenesis  • PPI hubs: *FZd5*. |
| **Considered but Excluded** | - Genes/miRNAs with inconsistent expression across datasets. - Biomarkers lacking functional annotations in diabetic nephropathy or acute coronary pathways (e.g., non-inflammatory genes). | • Example exclusions: Genes unrelated to angiogenesis or insulin resistance. |
| **Other public resources prioritized FZD5 and GTF2I**  **selection** | - Comparative Toxicogenomics Database - Gene Card database | Annotate **FZD5 and GTF2I** genes as linked to  angiogenesis **and** implicated in acute coronary syndrome a& diabetic nephropathy |
| **Previous Literature Validation** | Selected markers validated by prior studies (GTF2I & *FZd1* in diabetic cardiomyopathy). | • References: Supporting studies for GTF2I & *FZd1* in diabetic nephropathy or acute coronary.(references 23,24,25) |

**Supplementary Table 2:** Validation Metrics for Reference Genes.

| **Gene/Normalization** | **CV% (Inter-group)** | **ΔCt Range** | **Notes** |
| --- | --- | --- | --- |
| *SNORD44* | 5.2% | 1.1–1.8 | Small nucleolar RNA; minimal metabolic regulation |
| *Geometric Mean (GAPDH+ACTB)* | **4.9%** | **0.9–1.6** | Dual normalization reduces individual gene variability; MIQE-recommended strategy |
| *ACTB* | 6.8% | 1.3–2.1 | Validated in leukocytes for inflammatory conditions |
| *GAPDH* | 7.5% | 1.5–2.4 | Stable in chronic-phase samples (no acute glycemic shif |

| **Target symbol** | **Cat. No.** | **Gene Globe ID** | **Primer Assay** |
| --- | --- | --- | --- |
| **FZD5** | 249900 | QT00200886 | Hs_FZD5_1_SG QuantiTect Primer Assay |
| **GTF2I** |  | QT01677305 | Hs_GTF2I_2_SG QuantiTect Primer Assay |
| **GAPDH** |  | QT00079247 | Hs_GAPDH_1_SG QuantiTect Primer Assay |
| **ACTB** |  | QT00095431 | Hs_ACTB_1_SG QuantiTect Primer Assay |
| **Has-miR-1976** | 339350 | ZP00000388 | [hsa-miR-1976 miRCURY LNA miRNA Probe PCR Assay](https://geneglobe.qiagen.com/us/product-groups/mircury-lna-mirna-probe-pcr-assays/ZP00000388) |
| **SNORD44** | 339306 | YP00203902 | SNORD44 (hsa) miRCURY LNA miRNA PCR Assay |

**Supplementary Table 3:** All primer assays utilized in the study**.**

**Supplementary Table 4:** **Primer assay and PCR Efficiency for Target Genes.**

| **Gene** | **Gene globe ID** | **Amplicon Length (bp)** | **Annealing Temp (°C)** | **Efficiency (%)** |
| --- | --- | --- | --- | --- |
| **FZD5** | QT00200886 | 61 | 60 | 98 |
| **GTF2I** | QT01677305 | 77 | 58 | 102 |
| **miR-1976** | ZP00000388 | 52 | 62 | 92 |
| **LINC02210** | UPFH0591897 | 187 | 59 | 95 |
